# The influences of environmental change and development on leaf shape in *Vitis*

**DOI:** 10.1002/ajb2.1460

**Published:** 2020-04-09

**Authors:** Aly Baumgartner, Michaela Donahoo, Daniel H. Chitwood, Daniel J. Peppe

**Affiliations:** ^1^ Terrestrial Paleoclimate Research Group Department of Geosciences Baylor University Waco TX USA; ^2^ Department of Geological Sciences Texas Christian University Fort Worth TX USA; ^3^ Department of Horticulture Michigan State University East Lansing MI USA; ^4^ Department of Computational Mathematics, Science, and Engineering Michigan State University East Lansing MI USA

**Keywords:** paleoclimate proxy, phenotypic plasticity, leaf physiognomy, leaf ontogeny, climatic sensitivity

## Abstract

**Premise:**

The size and shape (physiognomy) of woody, dicotyledonous angiosperm leaves are correlated with climate. These relationships are the basis for multiple paleoclimate proxies. Here we test whether *Vitis* exhibits phenotypic plasticity and whether physiognomy varies along the vine.

**Methods:**

We used Digital Leaf Physiognomy (DiLP) to measure leaf characters of four *Vitis* species from the USDA Germplasm Repository (Geneva, New York) from the 2012–2013 and 2014–2015 leaf‐growing seasons, which had different environmental conditions.

**Results:**

Leaf shape changed allometrically through developmental stages; early stages were more linear than later stages. There were significant differences in physiognomy in the same developmental stage between the growing seasons, and species had significant differences in mean physiognomy between growing seasons. Phenotypic plasticity was defined as changes between growing seasons after controlling for developmental stage or after averaging all developmental stages. *Vitis amurensis* and *V. riparia* had the greatest phenotypic plasticity. North American species exhibited significant differences in tooth area:blade area. Intermediate developmental stages were most likely to exhibit phenotypic plasticity, and only *V. amurensis* exhibited phenotypic plasticity in later developmental stages.

**Conclusions:**

Leaves have variable phenotypic plasticity along the vine. Environmental signal was strongest in intermediate developmental stages. This is significant for leaf physiognomic‐paleoclimate proxies because these leaves are likely the most common in leaf litter and reflect leaves primarily included in paleoclimate reconstructions. Early season and early developmental stages have the potential to be confounding factors but are unlikely to exert significant influence because of differential preservation potential.

Angiosperm leaves exhibit a dramatic diversity of sizes and shapes. Leaf shape is dynamic and changes across many scales, from the evolutionary timescales that differentiate species (Bailey and Sinnott, 1915, 1916; Schmerler et al., 2012), to phenotypic plasticity during the lifetime of a single plant (Royer et al., 2008, 2009; Royer, 2012b; Chitwood et al., 2015, 2016; McKee et al., 2019), to heteroblasty at consecutive nodes as a plant grows (Jones, 1999), to the allometric changes in a single leaf as it develops (Nicotra et al., 2011). Changes on evolutionary timescales often lead to predictable patterns of leaf size and shape (physiognomy). As examples, high proportions of toothed leaves are found in cold temperate regions (Bailey and Sinnott, 1915, 1916), leaves tend to be smaller in arid environments and larger in wetter environments (e.g., Webb, 1968; Wilf et al., 1998; Jacobs, 1999; Malhado et al., 2009; Wright et al., 2017), and leaves tend to be more round in cooler environments and more elliptical in warmer environments (Peppe et al., 2011; Schmerler et al., 2012).

These relationships between leaf physiognomy and climate in woody dicotyledonous angiosperms have been noted for over 100 years (Bailey and Sinnott, 1915, 1916) and have been used to develop proxies for reconstructing paleoclimate (e.g., Bailey and Sinnott, 1915, 1916; Wing and Greenwood, 1993; Wolfe, 1993; Wilf, 1997; Wilf et al., 1998; Jacobs, 1999, 2002; Gregory‐Wodzicki, 2000; Adams et al., 2008; Peppe et al., 2011). These patterns of leaf physiognomy and climate have been tested on regional and global scales (e.g., Wolfe, 1979; Wilf, 1997; Peppe et al., 2011, 2018). However, to understand the processes driving changes in leaf physiognomy on a global scale, we must first understand the underlying drivers at individual sites. In addition, it is important to understand how leaf physiognomy changes through growth and development because changes in this timing could affect our understanding of these global patterns. Changes in leaf physiognomy of individual species can be influenced by a variety of factors—such as phenotypic plasticity, allometry, and heteroblasty—which need to be tested to understand the drivers of physiognomic changes through leaf development and in response to climate.

## Phenotypic plasticity

Phenotypic plasticity is the ability for a genotype to change phenotype in response to environmental change, such as temperature or precipitation. Work by Royer et al. (2009) demonstrated that *Acer rubrum* (Sapindaceae) exhibited significant phenotypic plasticity during the lifetime of the plant when seeds were grown in a contrasting climate from their source. For example, seeds from Florida grown in Rhode Island produced trees with leaves with more and larger teeth than their siblings from Florida grown in Florida. However, not all species exhibit phenotypic plasticity. For example, a study by Royer et al. (2008) demonstrated that while *Acer rubrum* exhibits phenotypic plasticity in response to changing temperature, *Quercus kelloggii* (Fagaceae) was phenotypically invariant to changing temperature. Furthermore, species of the same genus may exhibit differences in temperature sensitivity (Royer et al., 2008; McKee et al., 2019) and the effects of temperature on leaf physiognomy are often species specific (McKee et al., 2019).

## Allometry

In addition to phenotypic plasticity, studies of leaf physiognomy must consider both allometric (differences in shape due to varying growth rates across an organ) and heteroblastic (differences in shape at successive nodes) influences on leaf shape (e.g., Chitwood et al., 2015, 2016; Spriggs et al., 2018). Leaf physiognomy can change dramatically through growth and development (Evans, 1972). For example, leaf teeth are often disproportionately large in young leaves (Baker‐Brosh and Peet, 1997; Feild et al., 2005). In the leaves of many dicotyledonous angiosperms, the maturation of xylem and phloem in the midrib and higher order venation proceeds from the lamina base to the tip (acropetal), while the maturation of smaller veins proceeds from the lamina tip to the base (basipetal) (Turgeon, 1989). The different directions of maturation lead to variation in expansion rates in different regions of the leaf (allometric change). Furthermore, these patterns of allometric expansion are variable between species during leaf development (Das Gupta and Nath, 2015).

## Heteroblasty

Heteroblasty is the difference in the shape of leaves from successive nodes due to shoot apical meristem development (Ashby, 1948; Jones, 1993, 1999; Poethig, 2010; Zotz et al., 2011). Heteroblastic differences can range from subtle to striking and are affected by both physiological and environmental factors (Poethig, 1990; Gould, 1993; Day et al., 1997; Kerstetter and Poethig, 1998; Winn, 1999; Darrow et al., 2001; Chitwood et al., 2012). A drastic example is *Pseudopanax crassifolius* (Araliaceae) of New Zealand, which exhibits dramatically different leaves throughout its growth (Gould, 1993). The differences in leaf physiognomy in *P. crassifolious* are related to different drivers of leaf shape through plant growth and are correlated with different tree heights. Therefore, as the tree grows, leaves from consecutive nodes differ in shape due to the different physiological and environmental drivers of leaf shape (i.e. leaf construction costs, light interception, heat dissipation, etc.). Heteroblasty is uncommon in trees and shrubs, particularly those with deciduous habit (Dawson and Lucas, 2005), but is widespread on islands such as New Zealand and New Caledonia (Burns and Dawson, 2006). In vines and lianas, heteroblasty is much more common (Zotz et al., 2011) and differences in leaf physiognomy at consecutive nodes is due to a combination of both allometry and heteroblasty.

Phenotypic plasticity and heteroblasty can be easily confused and the terms are often used interchangeably (Diggle, 2002). Phenotypic plasticity refers to differences in leaf shape due to changing environmental conditions during the lifetime of a plant, while heteroblasty is a feature of shoot growth but is also regulated by environmental factors (Kerstetter and Poethig, 1998). However, it can be difficult to differentiate changes in leaf shape due to environmental phenotypic plasticity, allometric changes, or heteroblasty (Coleman et al., 1994).

## Leaf physiognomy of *Vitis*

*Vitis* (Vitaceae) is an economically important genus of temperate woody vines. The leaves of *Vitis* can range from simple to nearly compound, with shapes varying from orbicular to reniform to cordate (Chitwood et al., 2014, 2015, 2016). Previous work by Chitwood et al. (2016) analyzed >5500 leaves from multiple species of *Vitis* and *Ampelocissus* (Vitaceae). Their study showed that a more pronounced distal sinus, independent of species or developmental stage, was associated with colder and drier conditions. *Vitis* is an ideal taxon in which to assess the effects of phenotypic plasticity, allometry, and heteroblasty because its leaf physiognomy is influenced by all three factors.

In this study we address the following three questions to test that leaf shape responds to changes in temperature and precipitation and that leaf shape scales through development: (1) How does *Vitis* leaf shape change along the vine? (2) Does *Vitis* leaf shape respond to environmental changes between growing seasons? (3) Do different species respond to changes in temperature and precipitation in the same ways? We tested the leaf physiognomic‐development and leaf physiognomic‐environment relationships in four species of *Vitis* collected in two growing seasons with different environmental conditions to address these questions.

## MATERIALS AND METHODS

### Leaf collection

**Figure 1:**
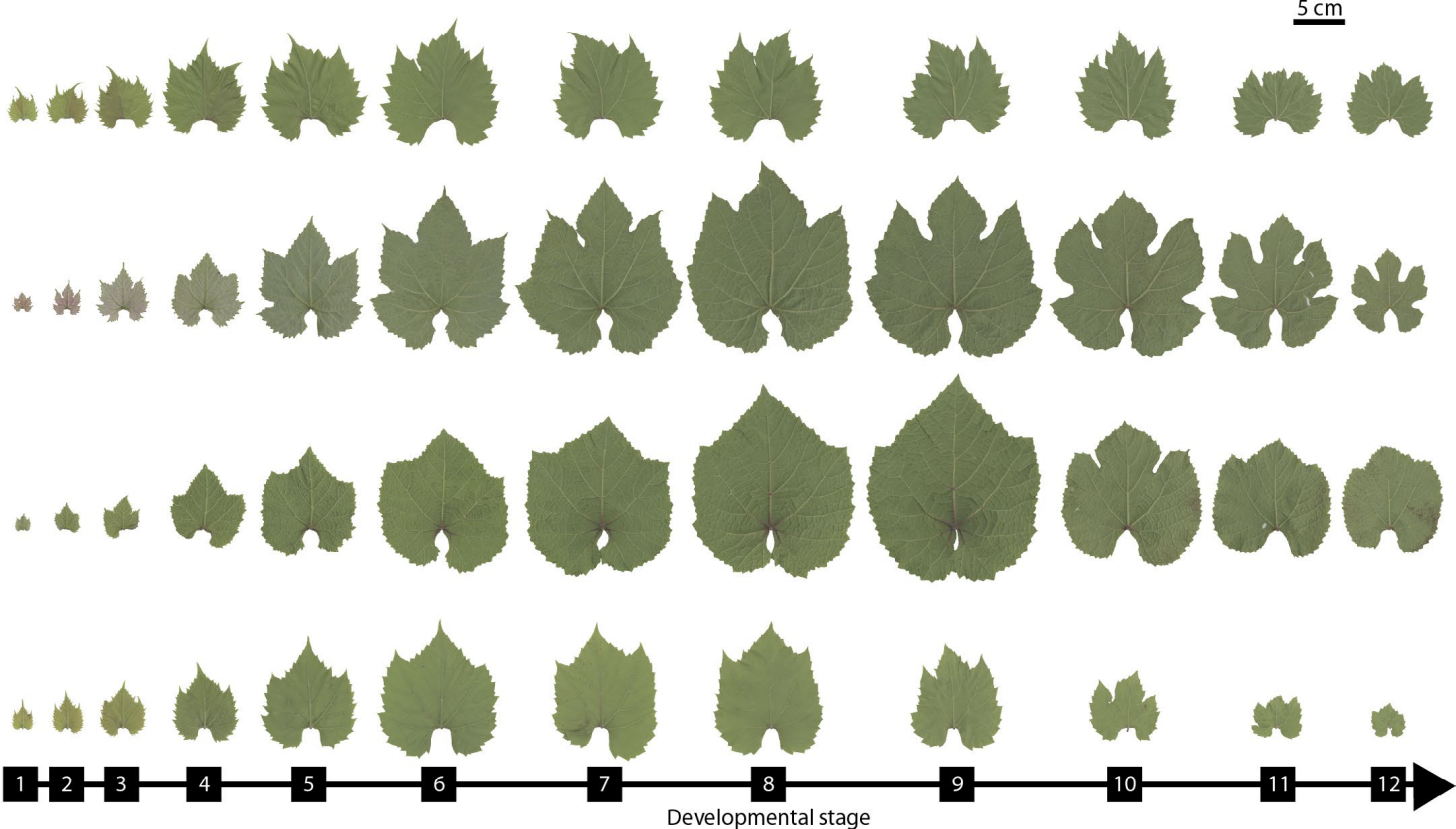
Four species of *Vitis*. From the top: *Vitis acerifolia*,* Vitis aestivalis*,* Vitis riparia*, and *Vitis amurensis*. Leaves are numbered from shoot tip (1) to base (12). Petioles are aligned to show differences in leaf size and shape between species and through development. The scale bar is 5 cm.

The leaves of four species of *Vitis* (*V. acerifolia*,* V. aestivalis*,* V. amurensis*, and *V. riparia*) were sampled from the USDA Germplasm Repository in Geneva, New York, USA by D. Chitwood (Fig. 1). The leaves used in this study were collected from the same vines during June 2013 and June 2015, and photographed for digital analysis. For a more detailed description of the collection process, see Chitwood et al. (2015, 2016).

The species used in this study (*V. acerifolia*,* V. aestivalis*,* V. amurensis*, and *V. riparia*) were selected because previous leaf morphometric research demonstrated that they represented a range of leaf shapes in *Vitis* and show differential changes through development (Chitwood et al., 2015, 2016). For example, Chitwood et al. (2015) found that the developmental stages of *V. riparia* and *V. acerifolia* were well predicted using a linear discriminant analysis, while the developmental stages of *V. aestivalis* were primarily underestimated. *Vitis amurensis* showed similar patterns to *V. riparia* and *V. acerifolia*, and was also selected because it is a phylogenetic outgroup to the other species used in this study.

In *Vitis*, leaf shape is patterned within buds during the year prior to budburst, therefore temperature and precipitation data were evaluated starting in the year before collection when temperatures began to rise above freezing until the time of collection (Morrison, 1991; Carmona et al., 2008; Chitwood et al., 2016). Here we used “leaf‐growing season,” which refers to roughly the period of time when leaf shape bud patterning began until leaf collection, which was an interval of approximately 15 months from March until June of the following year (methods of Chitwood et al., 2016). Following the methods of Chitwood et al. (2016), leaf wetness hours—which is influenced by precipitation in addition to other environmental factors including solar radiation, wind, and relative humidity—were used rather than mean annual precipitation because the pattern of precipitation was complicated (2014 had more precipitation than 2012, but 2013 had more precipitation than 2015). The two leaf‐growing seasons had different average temperature and leaf wetness hours: in 2012–2013 the mean temperature was 10.4°C with a mean of 5.6 leaf wetness hours per day, and in 2014–2015 the mean temperature was 8.2°C with a mean of 3.8 leaf wetness hours per day. Temperatures were consistently higher during the 2012–2013 leaf‐growing season than 2014–2015 (2.2°C) and leaf wetness hours were higher during the 2012–2013 leaf‐growing season than 2014–2015 (1.8 leaf wetness hours). Thus, the 2012–2013 leaf‐growing season was generally warmer and wetter than 2014–2015.

Previous phylogenetic analysis of *Vitis* placed *V. acerifolia*,* V. aestivalis,* and *V. riparia* in distinct, but closely associated clades, while *V. amurensis* was a phylogenetically distinct group (Zecca et al., 2012). *Vitis acerifolia*,* V. aestivalis*, and *V. riparia* are native to eastern North America; *V. riparia* has the widest range and spans from southern Canada to the Gulf of Mexico and from the Rocky Mountains to the Atlantic Ocean. The collection area is within its native geographic range. *Vitis aestivalis* spans from the northern United States to southern Texas and from the Great Plains to the Atlantic Ocean, with the collection site being within its native geographic range. *Vitis acerifolia* spans from the central Great Plains to northern Mexico and from the western Great Plains to the Gulf of Mexico. The collection area for this species is outside of its native geographic range. The native ranges of these three species overlap in northern Texas and southern Oklahoma. *Vitis amurensis* is native to East Asia and spans from the Amur Valley of Russia to the Korean Peninsula and from eastern China to Japan. Thus, the collection site is far outside the native geographic range of this species. The mean annual temperature (MAT) and mean annual precipitation (MAP) of the collection site in Geneva, New York are similar to those of the native range of *V. amurensis* (MAT: 9.8°C; MAP: 684 mm/y); however, the mean annual range of temperatures is notably smaller (Qian and Ricklefs, 2004; Fang et al., 2011). In addition, species native to eastern North America tend to have shorter vegetation periods than their European and East Asian relatives, meaning that the leaves flush later and senesce earlier (Zohner and Renner, 2017).

### Digital leaf physiognomy

**Figure 2:**
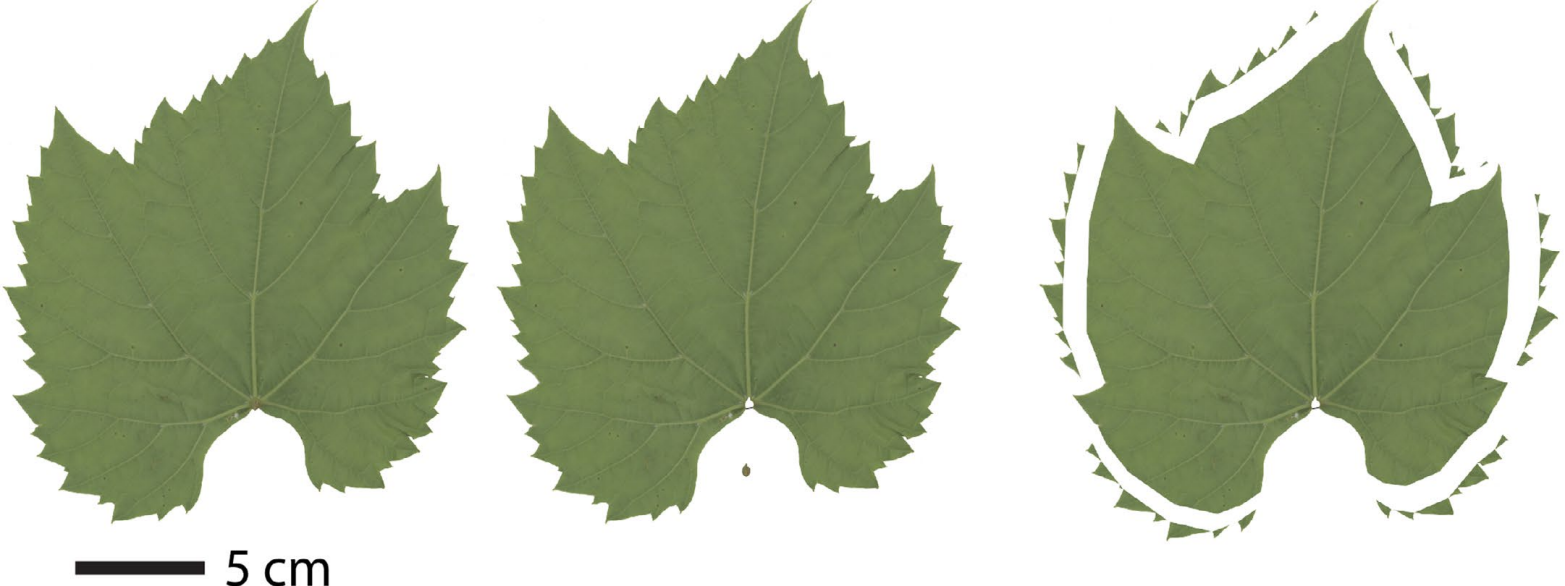
A *Vitis acerifolia* leaf processed with the Digital Leaf Physiognomy method. The petiole is removed to measure blade area. Internal perimeter is the perimeter of the leaf with the teeth removed.

Leaves were measured using the Digital Leaf Physiognomy (DiLP) protocol (Huff et al., 2003; Royer et al., 2005; Peppe et al., 2011) (Fig. 2). Images were prepared in Adobe Photoshop 8.0 (Adobe Systems Inc., San Jose, California, USA). Damaged leaf margins were reconstructed with a straight line; damaged or missing teeth were not reconstructed. When possible, the petiole was removed with a straight line cut at the intersection between the leaf lamina and the petiole. Lobes were defined as vascularized extensions of the margin supported by primary venation. Primary teeth were defined as a vascularized extension of the margin supported by secondary venation, and secondary teeth were defined as a vascularized extension of the margin supported by tertiary venation. So that we could systematically analyze all leaves between species and development stages in the same way, these definitions for lobes and teeth are modified slightly from the definitions of Ellis et al. (2009). Following the rules of Royer et al. (2005), leaf teeth were removed with a straight line cut from sinus to sinus, and secondary teeth were treated as extensions of the superjacent primary tooth (Fig. 2). Digital measurements were made using ImageJ (Abràmoff et al., 2004). Images were converted to grayscale and the threshold was adjusted so the leaf was a solid color and distinct from the background. Blade area, Feret diameter, major Feret diameter, perimeter, internal perimeter, primary teeth, secondary teeth, total teeth, and tooth area were measured, and all other variables were calculated from these measurements (Appendix S1).

### Morphometric analysis, statistics, and visualization

In an effort to differentiate between phenotypic plasticity and allometric developmental changes, ratios of leaf characters were used to correct for differences in leaf size when comparing different species and developmental stages. The following ratios were used: Feret diameter ratio, tooth area:perimeter, tooth area:internal perimeter, tooth area:blade area, total teeth:perimeter, total teeth:internal perimeter, total teeth:blade area, perimeter:area, perimeter ratio, compactness, and shape factor (Appendix S1). Total teeth, leaf area and average tooth area were also used to compare changes in leaf shape.

In this study, developmental stage was measured from vine tip to vine base; developmental stage 1 is the youngest and leaf age increases with developmental stage (see Chitwood et al., 2016; Fig. 1). Developmental stage was used instead of leaf number for consistency because in some species the number of leaves varied greatly between vines. In addition, the focus of this project was developmental rather than heteroblastic differences in leaf shape, so we prioritized grouping based on developmental stage rather than homologous node. However, this means that physiognomic differences between developmental stages are due to a combination of allometric and heteroblastic changes. Analyses were limited to the highest developmental stage that was present for both leaf‐growing seasons and had a combined total from both leaf‐growing seasons of at least five leaves. *Vitis acerifolia* and *V. aestivalis* were limited to stages 1–12, *V. amurensis* was limited to stages 1–13, and *V. riparia* was limited to stages 1–14 (Fig. 1 and Appendix S2). For *V. acerifolia*, 11 plants were sampled with 101 leaves analyzed from 2013, and 102 leaves analyzed from 2015 (Table 1 and Appendix S2). For *V. aestivalis*, six plants were sampled with 49 leaves analyzed from 2013, and 45 leaves analyzed from 2015. For *V. amurensis*, 15 plants were sampled with 143 leaves analyzed from 2013, and 140 leaves analyzed from 2015. For *V. riparia*, 17 plants were sampled with 173 leaves analyzed from 2013, and 142 leaves analyzed from 2015.

**Table 1:** Plant and leaf totals from each species for 2013 and 2015.

Species	Plants	2013	2015
*V. acerifolia*	11	101	102
*V. aestivalis*	6	49	45
*V. amurensis*	15	143	140
*V. riparia*	17	173	142

Bootstrap forest analyses (JMP^®^, Version 12.0. SAS Institute Inc., Cary, North Carolina, USA) were performed to determine whether the different *Vitis* species could be differentiated based on the measured leaf characters, and if the leaves of each species could be differentiated by year. Student's t‐tests were performed in R (base package) to determine whether leaf shape characters changed between leaf‐growing seasons. In an effort to differentiate between phenotypic plasticity and allometric developmental changes, linear modeling (base package) and breakpoint analysis (‘segmented’; Muggeo, 2008) were performed to determine whether there were consistent developmental “bins” of distinct leaf shapes. Principal component analysis (‘missMDA’; Josse and Husson, 2016) was performed for each species to determine which characters were the primary drivers of physiognomic variability and whether the developmental bins clustered together (Appendices [Link], [Link], [Link], [Link], [Link], [Link], [Link], [Link]). Principal component analysis was also performed on all species to determine whether the four species could be differentiated based on the principal components (Appendices S11 and S12).

Once bins were determined, physiognomic characters that were significantly different between the two leaf‐growing seasons were analyzed separately. Additional student's t‐tests were run to determine whether changes in physiognomy were consistent through all bins. The changes between leaf‐growing seasons were compared to the correlation table in Peppe et al. (2011) to determine whether the directionality of changes in leaf shape were as expected for the known changes in temperature and precipitation between the leaf‐growing seasons in 2012–2013 and 2014–2015 (i.e., temperature and precipitation decreased) (Appendix S1). Finally, changes within developmental bins were compared to species means to determine whether developmental changes were reflected in the mean. This was done to test whether changing the developmental composition of an assemblage—such as having an abundance of early or late developmental stages—would have an effect on the species mean. Because species means are used in paleoclimate analyses, it is important to test whether means reflect the changes along the vine.

## RESULTS

Bootstrap forest analysis showed that all four *Vitis* species could be distinguished based on physiognomic characters (Appendix S13); the misclassification rate was 5.21% (Appendix S14). Thus, all additional analyses were performed on each species individually. Bootstrap forest analysis also showed that for all species, leaf‐growing seasons were distinguishable based on the measured physiognomic characters (Appendices [Link], [Link]); the misclassification rate ranged from 4.10–8.77% (Appendix S15). There was considerable overlap in the principal components when comparing between species (Appendix S11 and S12).

Physiognomic characters changed along the vine. Many of the patterns of physiognomic change were parabolic and may reflect a combination of allometric and heteroblastic influences. Some characters were more sensitive to allometric and heteroblastic changes than others, and some characters demonstrated greater differences between leaf‐growing seasons than others. For example, Feret diameter ratio increased with developmental stage, meaning that leaves in earlier developmental stages were slightly more linear, while leaves in later developmental stages were nearly round. Characters related to the total number of teeth (total teeth:perimeter, total teeth:internal perimeter, and total teeth:blade area) were highly variable in the earlier developmental stages but were conserved in later developmental stages. Perimeter:area and compactness were also most variable in the early developmental stages but were conserved in later developmental stages. Perimeter ratio and shape factor had inverse relationships with developmental stage, and perimeter ratio decreased with developmental stage while shape factor increased with developmental stage. Tooth area:perimeter and tooth area:internal perimeter appeared to be driven by changes in tooth area, and tooth area:blade area was relatively stable in *V. acerifolia* and *V. riparia* but highly variable in *V. aestivalis* and *V. amurensis*.

**Figure 3:**
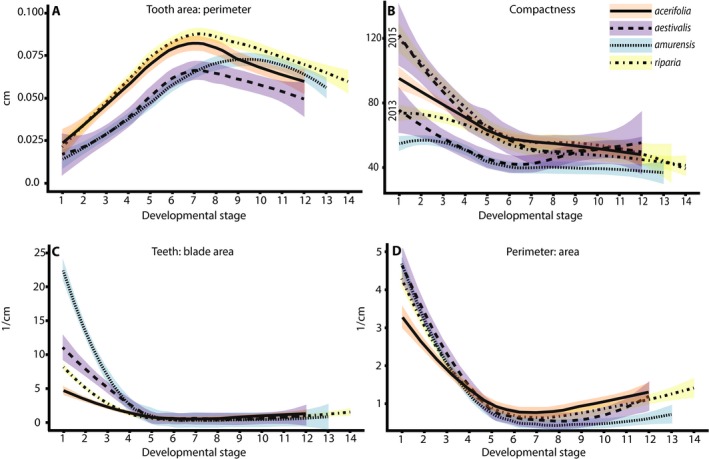
Leaf characters in four species of *Vitis* exhibited four general patterns. Both leaf‐growing seasons were combined when there were no significant statistical differences between leaf‐growing seasons for the character. (A) The developmental stages of *V. acerifolia*,* V. aestivalis*, and *V. riparia* followed the same trend, while *V. amurensis* values were offset, as demonstrated by tooth area: perimeter. (B) The developmental stages of some species had statistical differences between leaf‐growing seasons and different species clustered together based on year, as demonstrated by compactness. (C) The early developmental stages of all species were distinct, but late developmental stages were virtually indistinguishable, as demonstrated by total teeth: blade area. (D) The developmental stages showed no difference between leaf‐growing seasons and all species showed similar changes through development. Patterns of leaf characters (A) were similar for North American species but not *V. amurensis*, (B) were different between leaf‐growing seasons, (C) changed through development, or (D) were identical in all species.

There were four general patterns of leaf shape change (Fig. 3). First, in *V. acerifolia*,* V. aestivalis*, and *V. riparia* the average tooth area, tooth area:perimeter, and tooth area:internal perimeter increased with increasing developmental stage until developmental stage 7 and then decreased with increasing developmental stage, while in *V. amurensis* the peak occurred at developmental stage 9 (Fig. 3A). Similarly, in all species, Feret diameter increased with increasing developmental stage, but in *V. acerifolia, V. aestivalis,* and *V. riparia* the values completely overlapped, while in *V. amurensis* the values were higher than the other species. Second, for tooth area:blade area, perimeter ratio, compactness, and shape factor some species had statistical differences between leaf‐growing seasons and these different species clustered together based on leaf‐growing season (Fig. 3B). For tooth area: blade area, perimeter ratio, and compactness, the values increased from the 2012–2013 leaf‐growing season to the 2014–2015 leaf‐growing season; for shape factor the values decreased from the 2012–2013 leaf‐growing season to the 2014–2015 leaf‐growing season. Third, for total teeth:blade area, total teeth:perimeter, and total teeth:internal perimeter the values decreased with increasing developmental stage and the earliest developmental stages of all species were distinct, but later developmental stages were virtually indistinguishable (Fig. 3C). Finally, for total teeth and perimeter:area there was no obvious pattern between leaf‐growing seasons and all species had similar changes along the vine (Fig. 3D). Thus, leaf shape was plastic and changed between developmental stages of a single species from a single leaf‐growing season (allometric change and heteroblasty), as well between leaves of a single species of the same developmental stage between leaf‐growing seasons (phenotypic plasticity).

Because species were distinguishable by leaf‐growing season, we used student's t‐tests to determine which aspects of leaf shape were plastic. All four species of *Vitis* had statistically significant differences between leaf‐growing seasons for at least one leaf shape character: *V. acerifolia* showed differences in tooth area:blade area, *V. aestivalis* showed differences in total teeth, tooth area:blade area, and compactness, *V. amurensis* showed differences in total teeth, perimeter ratio, average tooth area, tooth area:perimeter, and tooth area:internal perimeter, and *V. riparia* showed differences in perimeter ratio, total teeth:perimeter, total teeth:internal perimeter, tooth area:blade area, tooth area:internal perimeter, compactness, and shape factor **(**Appendices [Link], [Link], [Link], [Link]). All of the North American species (*V. acerifolia*,* V. aestivalis*, and *V. riparia)* had statistical differences for tooth area:blade area, but *V. amurensis* did not.

Because there were notable changes in leaf shape characters along the vine, we used linear modeling to determine how much of the variance in leaf characters could be explained by developmental stage (allometric and heteroblastic changes). The linear relationship between leaf shape and developmental stage varied widely between the different species, although for all species, more than 30% of the variance in compactness and shape factor could be explained by developmental stage; in *V. aestivalis* this was only the case for the 2014–2015 leaf‐growing season (Appendices S21, S23, S25, S27). When there were dramatic differences in the variance between leaf‐growing seasons, it would often be much higher in the 2014–2015 leaf‐growing season, but not the reverse. We then used breakpoint analysis to determine at which developmental stages changes in leaf shape occurred. This analysis showed three leaf forms: a distinct early developmental form (early), a transitional form (intermediate), and a distinct fully developed form (late). The actual breakpoint differed for each leaf shape character and for each species (Appendices S22, S24, S26, S28); however, generally the early bin was stages 1–2, the intermediate bin was stages 3–10, and the late bin was stages 11–12 (Table 2)**.** Principal components analysis of each species clustered by developmental stage emphasized the parabolic nature of leaf shape (Appendices [Link], [Link], [Link], [Link], [Link], [Link], [Link], [Link]).

**Table 2:** Breakpoint analysis of *Vitis acerifolia, Vitis aestivalis, Vitis amurensis*, and *Vitis riparia* based on all measured leaf shape characters.

Species	Early bin	Intermediate bin	Late bin
*V. acerifolia*	1	2–11	12
*V. aestivalis*	1	2–10	11–12
*V. amurensis*	1–2	3–10	11–13
*V. riparia*	1–2	3–11	12–14

**Figure 4:**
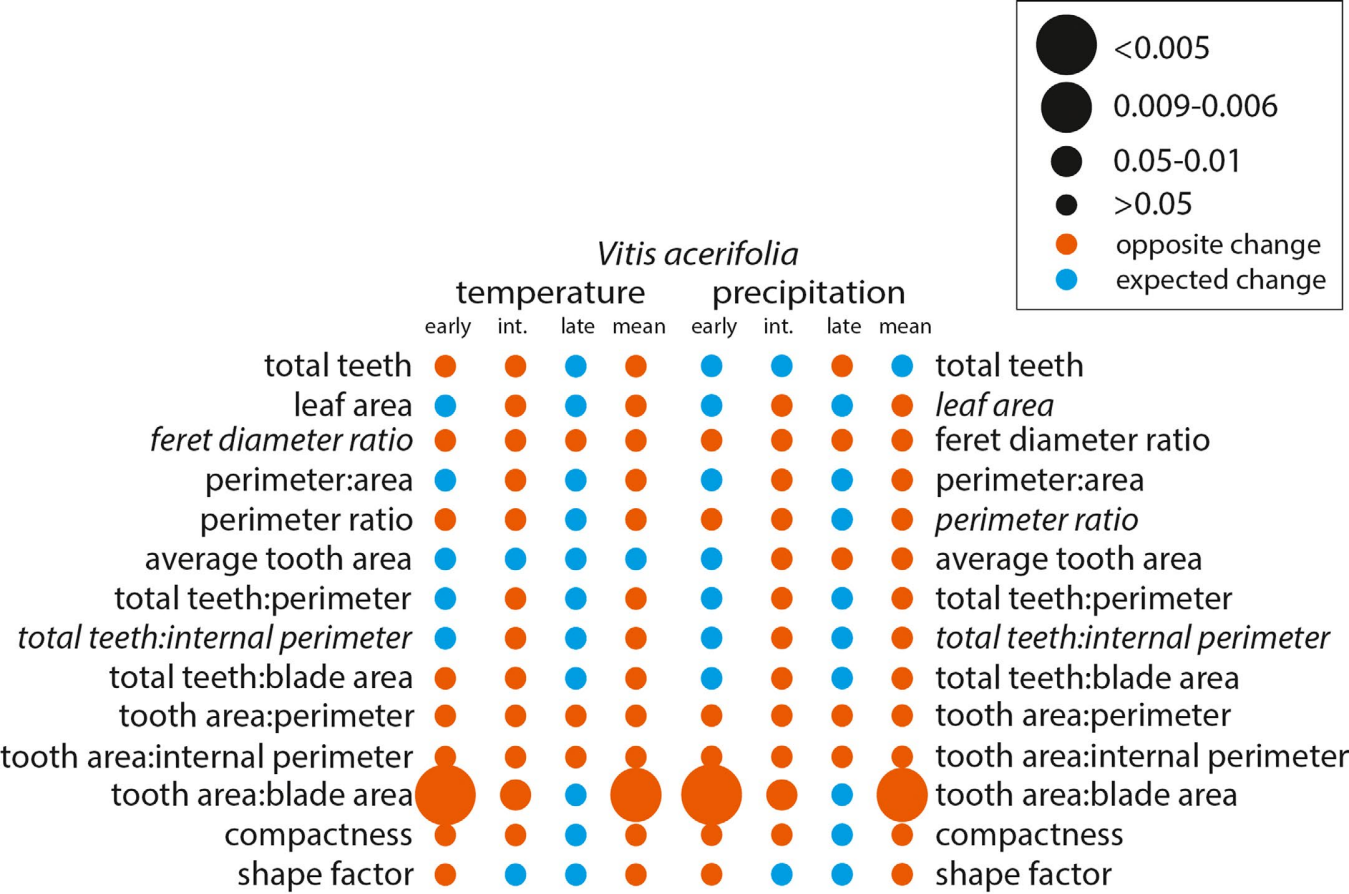
*Vitis acerifolia* binned significant effect table for temperature and precipitation. Leaf shape variables included in the Digital Leaf Physiognomy climate equation are italicized—the larger the circle, the stronger the statistical relationship. Circle color is determined by whether the change between leaf‐growing seasons was as expected based on the correlation in Peppe et al. (2011) (blue) or the opposite of the expected change (orange).

**Figure 5:**
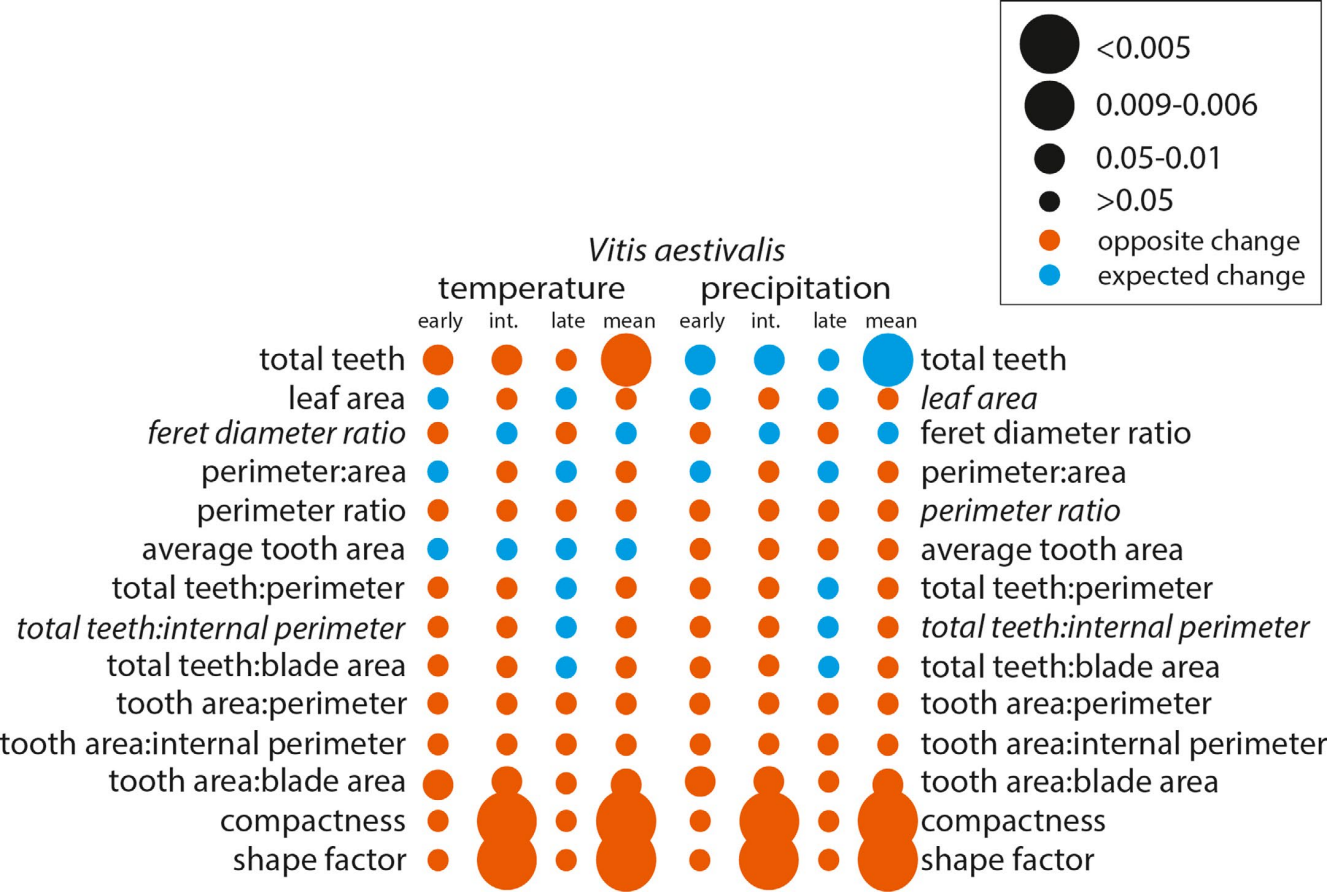
*Vitis aestivalis* binned significant effect table for temperature and precipitation. Leaf shape variables included in the Digital Leaf Physiognomy climate equation are italicized—the larger the circle, the stronger the statistical relationship. Circle color is determined by whether the change between leaf‐growing seasons was as expected based on the correlation in Peppe et al. (2011) (blue) or the opposite of the expected change (orange).

**Figure 6:**
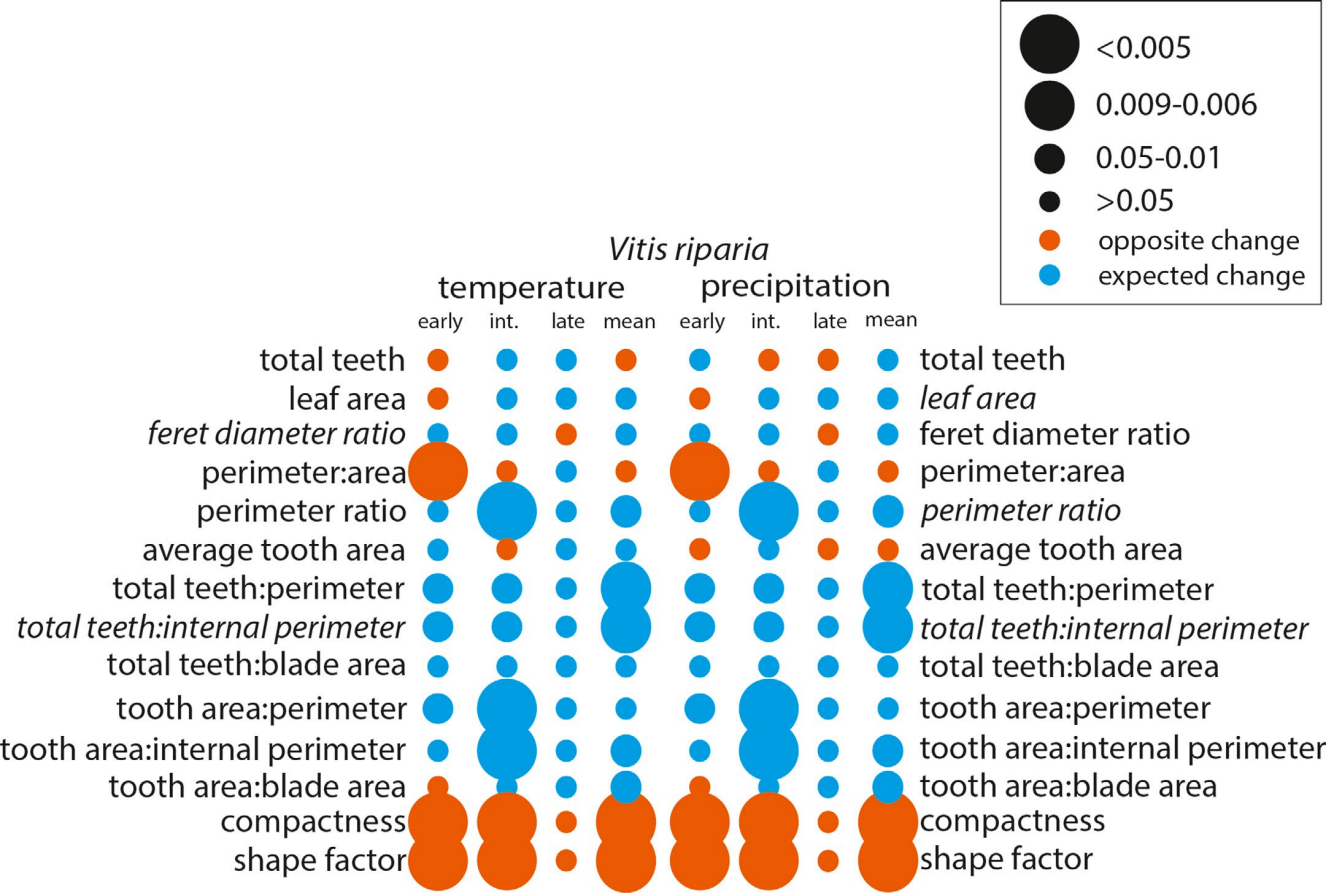
*Vitis riparia* binned significant effect table for temperature and precipitation. Leaf shape variables included in the Digital Leaf Physiognomy climate equation are italicized—the larger the circle, the stronger the statistical relationship. Circle color is determined by whether the change between leaf‐growing seasons was as expected based on the correlation in Peppe et al. (2011) (blue) or the opposite of the expected change (orange).

**Figure 7:**
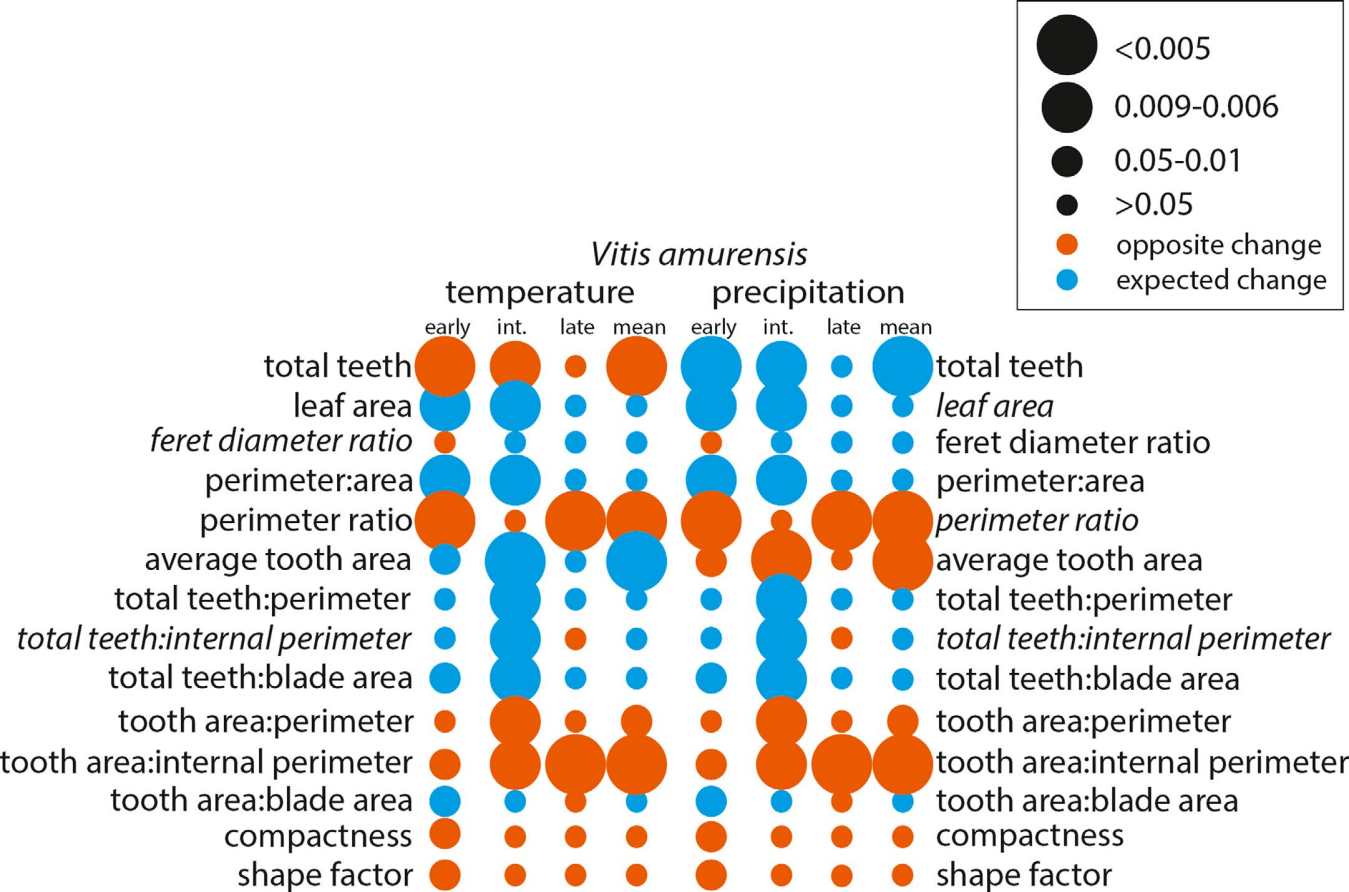
*Vitis amurensis* binned significant effect table for temperature and precipitation. Leaf shape variables included in the Digital Leaf Physiognomy climate equation are italicized. The larger the circle, the stronger the statistical relationship. Circle color is determined by whether the change between leaf‐growing seasons was as expected based on the correlation in Peppe et al. (2011) (blue) or the opposite of the expected change (orange).

We then used these developmental bins and the species means to determine whether changes between leaf‐growing seasons were due to the overrepresentation of a developmental bin, or large‐scale differences across bins due to phenotypic plasticity. For example, the overrepresentation of the early bin may decrease the Feret diameter ratio, which would also suggest a warming trend, or decrease the size, which would suggest a drying trend. The species showed different responses to the leaf‐growing seasons. *Vitis acerifolia* and *V. aestivalis* showed little phenotypic plasticity, but all changes in leaf shape were present in the species mean as well as either the intermediate bin or both the early and intermediate bins (Figs. 4 and 5). In contrast, *V. amurensis* and *V. riparia* showed considerable plasticity between leaf‐growing seasons, but not all changes in leaf shape were present in the species means. In *V. riparia*, most of the changes in leaf shape were present in the species mean as well as either the intermediate bin or both the early and intermediate bins with the exception of perimeter:area, which only changed in the early bin, and tooth area:blade area, which only changed in the species mean (Fig. 6). In *V. amurensis*, some of the changes in leaf shape were present in the species mean as well as either the intermediate bin or both the early and intermediate bins but many were not. Some measurements changed in one or more developmental bin but not the species mean. In addition, *V. amurensis* was the only species with significant changes in the late bin (Fig. 7).

After assessing for significant changes in leaf shape between leaf‐growing seasons, we assessed how the leaf characters changed to determine if these differences were consistent with our expectations of phenotypic plasticity in response to changes in temperature and precipitation (Appendix S1). For all species, the direction of change of the species mean for each leaf shape character reflected the significant correlations of each of the developmental bins. *Vitis acerifolia* and *V. aestivalis* were not sensitive to environmental variability. In *V. acerifolia* only tooth area:blade area varied between leaf‐growing seasons, but it did not change as expected with respect to temperature or precipitation (Fig. 4). In *V. aestivalis* tooth area:blade area, compactness, and shape factor varied between leaf‐growing seasons, but did not change as expected with respect to temperature or precipitation; total teeth changed as expected with respect to precipitation but not temperature (Fig. 5). *Vitis amurensis* and *V. riparia* were sensitive to environmental variability. In *V. amurensis*, perimeter ratio, tooth area:perimeter, and tooth area:internal perimeter varied between leaf‐growing seasons but did not change as expected with respect to temperature or precipitation. In addition, average tooth area changed as expected with respect to temperature but not precipitation and total teeth changed as expected with respect to precipitation but not temperature (Fig. 7). In *V. riparia*, compactness and shape factor varied between leaf‐growing seasons but did not change as expected with respect to temperature or precipitation, while perimeter ratio, total teeth: perimeter, total teeth:internal perimeter, tooth area:internal perimeter, and tooth area:blade area changed as expected with respect to both temperature and precipitation (Fig. 6).

## DISCUSSION

### Allometric and heteroblastic physiognomic change

Studies of leaf physiognomy must consider both allometric and heteroblastic influences on leaf shape (Chitwood et al., 2015, 2016). In addition to temperature and precipitation, the age of the vines changed between the leaf‐growing seasons. However, because *Vitis* is a long‐lived woody perennial, differences in leaf shape due to age of the plant were negligible compared to differences due to temperature and precipitation. Leaves were smaller at the base and tip of the shoot (Fig. 1). The earliest developmental stages—at the tip of the shoot—were relatively small because they were still undergoing allometric expansion and had not yet fully developed. The oldest leaves—at the base of the shoot—had reached maturity and were no longer expanding but were smaller than many of the younger leaves. Previous work by Chitwood et al. (2015) has shown that the diminutive size of these first‐formed leaves is genetically specified.

In addition, the growth habit of *Vitis* allows us to see a snapshot of allometric changes, but also emphasizes heteroblastic differences. Differences in leaf shape give us a physiognomic roadmap of allometric changes (Fig. 1). Previous studies of the relationship between bud packing and leaf shape have shown that “preformed” leaves, which undergo a period of arrested development due to overwintering in the bud, tend to differ from “neoformed” leaves, which are generally formed later in the season and therefore do not overwinter in a bud, but instead develop directly from leaf primordia (Critchfield, 1971; Edwards et al., 2016; Spriggs et al., 2018). For example, in *Viburnum* (Adoxaceae) preformed leaves tend to be rounder or more lobed and toothier than neoformed leaves, which tend to be more elliptical with reduced teeth (Edwards et al., 2016; Spriggs et al., 2018). *Vitis* exhibited a similar pattern. The early developmental stages—which formed later in the season—have long narrow teeth, which gives the leaf an overall more linear shape. In later developmental stages, the teeth become larger and more triangular. In addition, later developmental stages have more pronounced basal lobes, which gives the leaf a more circular shape. Depending on the species of *Vitis*, buds contain 5–7 leaf primordia (Dr. Jason Londo, USDA‐ARS Grapevine Genetics Research Unit, Geneva, New York, personal communication). These patterns of changes in leaf physiognomy show that in *Vitis*, leaves change allometrically through development, and heteroblastically due to bud packing.

Allometric and heteroblastic changes in leaf shape were affected by changing temperature and precipitation. For example, *V. acerifolia* only exhibited differences in leaf shape between leaf‐growing seasons for tooth area:blade area. However, this difference was most pronounced in the earliest developmental stages and was either less pronounced or entirely absent in the later developmental stages (Fig. 4). In *V. amurensis*, differences in tooth area:blade area, compactness, and shape factor were only significant in the early developmental stages (Fig. 7). It is worth noting that, with the exception of *V. amurensis*, the oldest leaves were invariant and not sensitive to changes in temperature or precipitation. This may mean that physiognomic changes in these developmental stages are not driven by temperature or precipitation. It is also important to consider that *V. amurensis* was cultivated far outside its native geographic range and it is clear from the pattern of developmental change in leaf shape characters, particularly those related to tooth area (average tooth area, tooth area:perimeter and tooth area:internal perimeter), that the timing of development differed from its North American relatives (Fig. 3A). This could be due to the shortened vegetation period in North America compared to East Asia (Zohner and Renner, 2017). However, *V. acerifolia* was also cultivated outside of its native geographic range and it does not exhibit this offset, but unlike *V. amurensis,* it is native to North America.

### Environmental physiognomic change

Previous work on *Vitis* by Chitwood et al. (2016) showed significant differences in the degree of dissection of the distal sinus between the 2012–2013 and 2014–2015 leaf‐growing seasons; they were able to match the leaf to the leaf‐growing season with 65.5–69.1% accuracy. The pronounced dissection of the distal sinus in the 2014–2015 leaf‐growing season is associated with cooler and drier growing conditions. If physiognomic sensitivity in *Vitis* were driven by temperature, we would expect the leaves from the 2012–2013 leaf‐growing season to have fewer, smaller teeth than the 2014–2015 leaf‐growing season. If physiognomic sensitivity were driven by precipitation, we would expect the leaves from the 2012–2013 leaf‐growing season to be larger and less dissected with a greater number of larger teeth than the 2014–2015 leaf‐growing season. Based on the correlations between the change in leaf shape and environment, these changes did not appear to be driven solely by temperature or precipitation, but a mixture of the two (Figs. 4, 5, 6, 7). Overall, differences in leaf shape between leaf‐growing seasons were primarily driven by tooth area and perimeter; to a lesser degree, differences were driven by the total number of teeth and internal perimeter. Leaf area did not have a strong influence on differences in leaf physiognomy. When compared to the correlations from Peppe et al. (2011), differences in characters related to the total number of teeth were often correlated as expected with precipitation, while differences related to tooth area were often correlated as expected with temperature.

*Vitis acerifolia*,* V. aestivalis*,* V. amurensis*, and *V. riparia* varied considerably in their phenotypic plasticity, however previous studies have shown that members of the same genus may have different degrees of phenotypic plasticity and that the effects of changing temperature on leaf physiognomy are species specific (Royer et al., 2008; McKee et al., 2019). Neither *V. acerifolia* nor *V. aestivalis* exhibited the expected change for either temperature or precipitation, while *V. riparia* exhibited the expected change for both. All three North American taxa exhibited significant differences in tooth area: blade area, although the functional explanation for this pattern is unclear.

*Vitis riparia* and *V. amurensis* were most sensitive to changes in temperature and precipitation, however each species had a different relationship between leaf shape and environment (Fig. 3). For example, both species had differences in perimeter:area in the youngest developmental stages, but *V. amurensis* exhibited the expected change for both temperature and precipitation, while *V. riparia* did not exhibit the expected change for either. Conversely, both species had differences in tooth area:internal perimeter in the majority of their leaves, but *V. riparia* exhibited the expected change for both temperature and precipitation while *V. amurensis* did not exhibit the expected change for either. Compactness and shape factor were plastic in *V. aestivalis*,* V. amurensis*, and *V. riparia* but no species exhibited the expected changes for either temperature or precipitation. This suggests that either *Vitis* has a different relationship between compactness and environment, and shape factor and environment or that changes in compactness and shape factor are influenced by something other than temperature or precipitation in *Vitis*.

It is important to note that *V. acerifolia*, which had the fewest statistically significant differences in leaf characters between leaf‐growing seasons, was the only species native to a warmer climate; *V. aestivalis* and *V. riparia* are both native to New York, USA. Although *V. amurensis* experiences similar temperatures in its native range in East Asia, East Asian winters tend to be much colder than those in eastern North America, while precipitation is generally higher in eastern North America than in East Asia (Qian and Ricklefs, 2004; Fang et al., 2011). *Vitis amurensis* is climatically sensitive but is growing outside of its native geographic range, which could explain why its leaf physiognomy did not change as expected with changing temperature and precipitation. It is also notable that *V. riparia*, which is the only species that was both climatically sensitive and growing in its native geographic range, had changes in physiognomy in response to environmental change that was almost entirely in the expected direction of the global data set (Peppe et al., 2011). *Vitis acerifolia* and *V. aestivalis* do not appear to be climatically sensitive, so their deviation from the expected correlation may be related to drivers other than changes in temperature or precipitation. These relatively climate insensitive responses may be due to the fact that *Vitis* is a liana, and lianas have been shown to have a weaker relationship with leaf margin state and climate than trees or shrubs (Royer et al., 2012). In addition, *Vitis*’ preference for lowland and riparian environments is another potential confounding factor that may influence the leaf physiognomic‐climate relationship (see discussion in Royer, 2012a).

The *Vitis* species used in this study had variable phenotypic plasticity along their vines, which could reflect the different roles of leaves as the vine grew through the leaf‐growing season. The oldest leaves were invariant, which may allow them to maximize early season productivity or be driven by genetic controls that limit the plasticity of leaf size and shape (Chitwood et al., 2015). Physiognomic changes in newly flushed leaves are driven by allometric expansion, however it is unclear from this study whether the degree of climate sensitivity in newly flushed leaves varies through the leaf‐growing season. In our analysis of the two species that showed environmental sensitivity—*V. amurensis* and *V. riparia*—leaves that had completed most of their allometric expansion but did not flush early in the growing season (i.e., the intermediate bin), showed the most phenotypic plasticity. Therefore, we interpret that the climate signal was strongest in these leaves.

Previous work on *Vitis* has shown that members of the genus are climatically sensitive (Chitwood et al., 2016). However, not all species of *Vitis* exhibited phenotypic plasticity in the variables used in the Peppe et al. (2011) DiLP paleoclimate equations (Feret diameter ratio and total teeth:internal perimeter for MAT and leaf area, perimeter ratio, and total teeth:internal perimeter for MAP). *Vitis acerifolia* and *V. aestivalis* did not have significant differences in any of the DiLP variables, and no species had any differences in Feret diameter ratio. *Vitis amurensis* had significant differences in perimeter ratio at the species mean level, and some developmental stages had significant differences in total teeth:internal perimeter and leaf area, although neither were significant at the species mean level. *Vitis riparia* only had significant differences of the species mean in total teeth:internal perimeter and perimeter ratio.

Interestingly, species means did not reflect the differences in phenotypic plasticity along the vine. For example, *V. riparia* exhibited small (though significant) differences in total teeth:perimeter and total teeth:internal perimeter in the early and intermediate bins, however the species mean showed large differences between years (Fig. 6). Similarly, there were no differences in tooth area:blade area in any bin but the species mean showed a small but significant difference. *Vitis amurensis* exhibited significant differences in total teeth:perimeter, total teeth:internal perimeter, and total teeth:blade area in the intermediate bin but none of these differences were significant at the species mean level (Fig. 7). This is important because leaf physiognomic paleoclimate methods rely on species means to estimate paleotemperature and paleoprecipitation, which suggests that a grand species mean may not fully reflect the variability within a taxon and may mask developmental variability. This is relevant to leaf physiognomic paleoclimate proxies because intermediate and late developmental stages are most likely to be preserved in leaf litter and reflect the type of leaves included in paleoclimate reconstructions (Burnham et al., 1992). Macrofloral fossil assemblages are typically composed of leaves that have fallen naturally, often from intermediate to late developmental stages. This suggests that while leaf development does have the potential to be a confounding factor, it is unlikely to exert a significant influence on analysis because of the unlikelihood of early developmental stages or early season leaves being preserved.

## CONCLUSIONS

Leaf physiognomic paleoclimate proxies assume that the leaves of woody dicotyledonous angiosperms change isometrically through development and reliably reflect temperature and precipitation. We found that the leaves of four species of *Vitis* changed allometrically through development and that leaves had variable phenotypic plasticity along the vine. This suggests that, at least in species that demonstrate phenotypic plasticity, leaves reflect the environmental conditions during bud patterning with the exception of the first flushed leaves, which are physiognomically invariant. In addition, the relationship between leaf shape and environmental signal was strongest in leaves that had completed allometric expansion and in taxa growing in their native range. Finally, leaf development has the potential to be a confounding factor in leaf physiognomic paleoclimate proxies, but it is unlikely to exert a significant influence on analysis due to differential preservation potential. This is significant because these leaves are most likely to be preserved in leaf litter and reflect the most common type of leaves included in paleoclimate reconstructions.

## AUTHOR CONTRIBUTIONS

A.B., D.C., and D.P. developed the project. D.C. collected the data, which was processed and analyzed by A.B. and M.D.; A.B., D.C., and D.P. developed the statistical analyses. A.B. wrote the manuscript with input from D.C., M.D., and D.P.

## Supporting information


**APPENDIX S1.** Definitions of physiognomic variables used in this study and the expected direction of change based on Peppe et al. (2011).

**APPENDIX S2.** Leaf totals from each species for 2013 and 2015.

**APPENDIX S3.** Principal components analysis of *V. acerifolia* clustered by developmental stage.

**APPENDIX S4.** Loadings of the principal components for the first five dimensions for *V. acerifolia*.

**APPENDIX S5.** Principal components analysis of *V. aestivalis* clustered by developmental stage.

**APPENDIX S6.** Loadings of the principal components for the first five dimensions for *V. aestivalis*.

**APPENDIX S7.** Principal components analysis of *V. riparia* clustered by developmental stage.

**APPENDIX S8.** Loadings of the principal components for the first five dimensions for *V. riparia*.

**APPENDIX S9.** Principal components analysis of *V. amurensis* clustered by developmental stage.

**APPENDIX S10.** Loadings of the principal components for the first five dimensions for *V. amurensis*.

**APPENDIX S11.** Principal components analysis of *Vitis* clustered by species.

**APPENDIX S12.** Loadings of the principal components for the first five dimensions for *Vitis*.

**APPENDIX S13.** Bootstrap Forest analysis of *Vitis* species based on leaf shape.

**APPENDIX S14.** Bootstrap Forest analysis of *Vitis* species based on all measured leaf shape characters.

**APPENDIX S15.** Bootstrap Forest analysis of *Vitis acerifolia*,* Vitis aestivalis*,* Vitis amurensis*, and *Vitis riparia* by year based on leaf shape.

**APPENDIX S16.** Bootstrap Forest analysis of *Vitis acerifolia, Vitis aestivalis, Vitis amurensis*, and *Vitis riparia* by year based on all measured leaf shape characters.

**APPENDIX S17.** Student's t‐tests of *Vitis acerifolia* of all measured leaf characters.

**APPENDIX S18.** Student's t‐tests of *Vitis aestivalis* of all measured leaf characters.

**APPENDIX S19.** Student's t‐tests of *Vitis amurensis* of all measured leaf characters.

**APPENDIX S20.** Student's t‐tests of *Vitis riparia* of all measured leaf characters.

**APPENDIX S21.** Linear model of *Vitis acerifolia* based on all measured leaf shape characters.

**APPENDIX S22.** Breakpoint analysis of *Vitis acerifolia* based on all measured leaf shape characters.

**APPENDIX S23.** Linear model of *Vitis aestivalis* based on all measured leaf shape characters.

**APPENDIX S24.** Breakpoint analysis of *Vitis aestivalis* based on all measured leaf shape characters.

**APPENDIX S25.** Linear model of *Vitis amurensis* based on all measured leaf shape characters. Bold text denotes R^2^ ≥ 0.3.

**APPENDIX S26.** Breakpoint analysis of *Vitis amurensis* based on all measured leaf shape characters.

**APPENDIX S27.** Linear model of *Vitis riparia* based on all measured leaf shape characters.

**APPENDIX S28.** Breakpoint analysis of *Vitis riparia* based on all measured leaf shape characters.

## Data Availability

The data set analyzed during the current study is available in the Texas Data Repository (https://dataverse.tdl.org/dataverse/VitisLeaf).
