# Supplementary material for: The influences of environmental change and development on leaf shape in Vitis
Source: Am J Bot. 2020 Apr 9;107(4):676–88. doi: 10.1002/ajb2.1460 (PMC7217169; doi:10.1002/ajb2.1460)
Supplement: Supplementary file 1 — APPENDIX S1. Definitions of physiognomic variables used in this study and the expected direction of change based on Peppe et al. (2011). [file AJB2-107-676-s001.pdf]

Appendix S1. Definitions of physiognomic variables used in this study and the expected direction of change based on Peppe et al. (2011).

Variable	Definition (units)	Expected direction of change
Blade area	Area of the leaf blade with petiole removed (cm)	Positive correlation with temperature and precipitation
Perimeter	Leaf blade perimeter (cm)	
Internal perimeter	Leaf blade perimeter with teeth removed (cm)	
Feret diameter	Diameter of a circle with the same area as the leaf (cm)	
Major feret	Longest measurable line across the leaf blade (cm)	
Primary teeth	Number of primary teeth (count)	
Secondary teeth	Number of secondary teeth (count)	
Total teeth	Number of primary and secondary teeth	Negative correlation with temperature, no significant correlation with precipitation
Tooth area	Area of teeth (cm^2)	
Average tooth area	Tooth area/number of primary teeth (cm^2)	No significant correlation with temperature or precipitation
Perimeter ratio	Perimeter/internal perimeter (dimensionless)	Negative correlation with temperature, no significant correlation with precipitation
Perimeter: Area	Perimeter/blade area	
Compactness	$Perimeter^2$/blade area (dimensionless)	Negative correlation with temperature, no significant correlation with precipitation
Shape factor	4π * blade area/$perimeter^2$ (dimensionless)	Positive correlation with temperature, no significant correlation with precipitation
Feret diameter ratio	Feret diameter/major feret (dimensionless)	Negative correlation with temperature, no significant correlation with precipitation
Total teeth: blade area	Number of teeth/blade area (cm^{-1})	
Total teeth: perimeter	Number of teeth/perimeter (cm^{-1})	Negative correlation with temperature, no significant correlation with precipitation
Total teeth: internal perimeter	Number of teeth/internal perimeter (cm^{-1})	Negative correlation with temperature, no significant correlation with precipitation
Tooth area: blade area	Tooth area/blade area (dimensionless)	Negative correlation with temperature and precipitation

Tooth area: perimeter	Tooth area/perimeter (cm)	Negative correlation with temperature, no significant correlation with precipitation
Tooth area: internal perimeter	Tooth area/ internal perimeter (cm)	Negative correlation with temperature, no significant correlation with precipitation
