# Supplementary material for: The influences of environmental change and development on leaf shape in Vitis
Source: Am J Bot. 2020 Apr 9;107(4):676–88. doi: 10.1002/ajb2.1460 (PMC7217169; doi:10.1002/ajb2.1460)
Supplement: Supplementary file 2 — APPENDIX S2. Leaf totals from each species for 2013 and 2015. [file AJB2-107-676-s002.pdf]

Appendix S2. Leaf totals from each species for 2013 and 2015.

**Species**	**Year**	**1**	**2**	**3**	**4**	**5**	**6**	**7**	**8**	**9**	**10**	**11**	**12**	**13**	**14**
V. acerifolia	2013	2	9	10	10	11	10	9	9	8	7	9	7		
	2015	5	10	11	10	10	10	11	9	9	8	5	5		
V. aestivalis	2013	1	3	4	4	5	5	6	5	6	4	3	4		
	2015	2	4	5	5	6	5	5	4	2	4	2	2		
V. amurensis	2013	13	13	14	13	12	11	12	9	9	7	10	11	10	
	2015	15	11	12	14	12	12	11	12	9	12	10	6	5	
V. riparia	2013	13	15	15	15	14	14	14	14	8	8	12	10	12	10
	2015	10	9	15	15	15	15	14	13	10	10	8	5	3	1
