# Supplementary figures and images for: The influences of environmental change and development on leaf shape in Vitis
Source: Am J Bot. 2020 Apr 9;107(4):676–88. doi: 10.1002/ajb2.1460 (PMC7217169; doi:10.1002/ajb2.1460)

Appendix S3. Principal components analysis of *V. acerifolia* clustered by developmental stage.

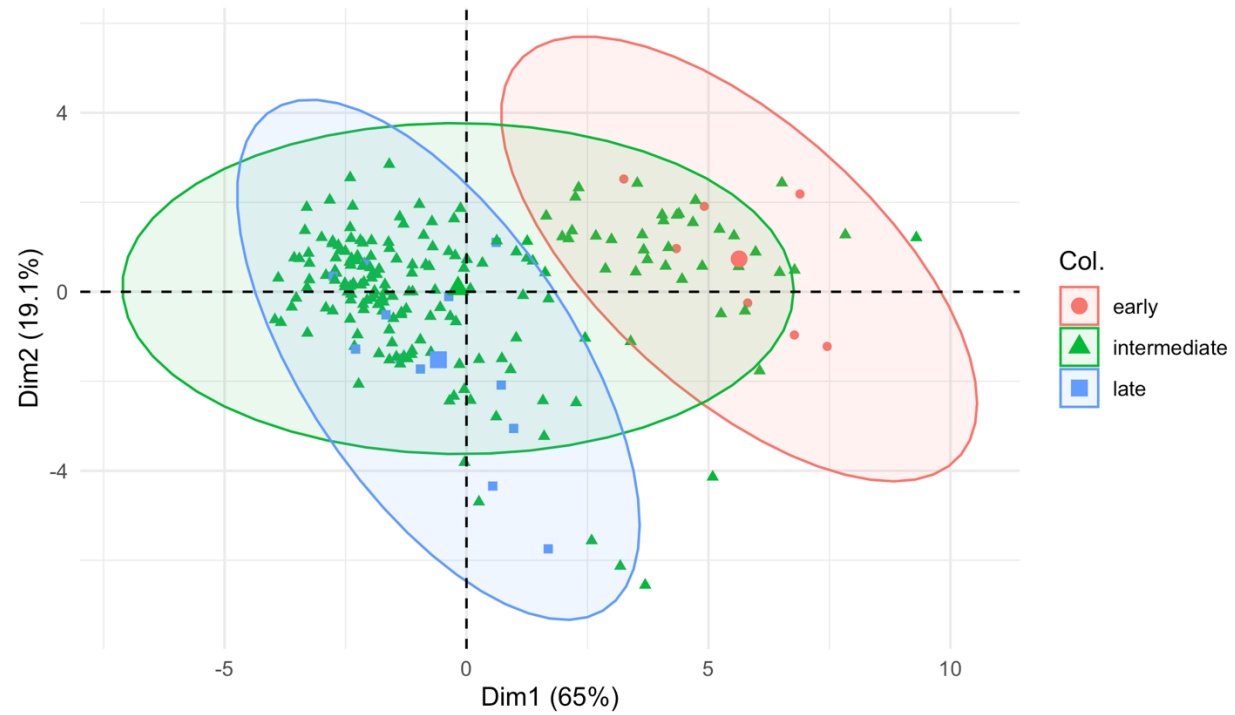

Supplement: Supplementary file 3 — APPENDIX S3. Principal components analysis of V. acerifolia clustered by developmental stage. [file AJB2-107-676-s003.pdf]

Appendix S5. Principal components analysis of *V. aestivalis* clustered by developmental stage.

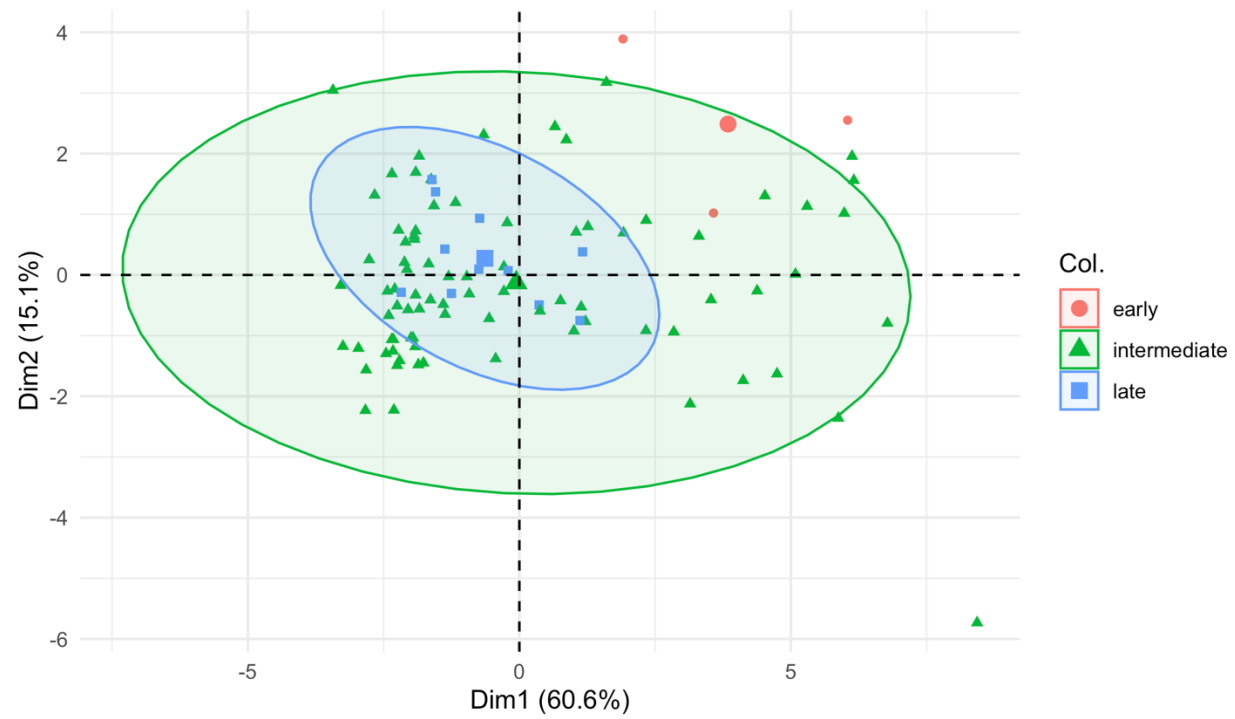

Supplement: Supplementary file 5 — APPENDIX S5. Principal components analysis of V. aestivalis clustered by developmental stage. [file AJB2-107-676-s005.pdf]

Appendix S7. Principal components analysis of *V. riparia* clustered by developmental stage.

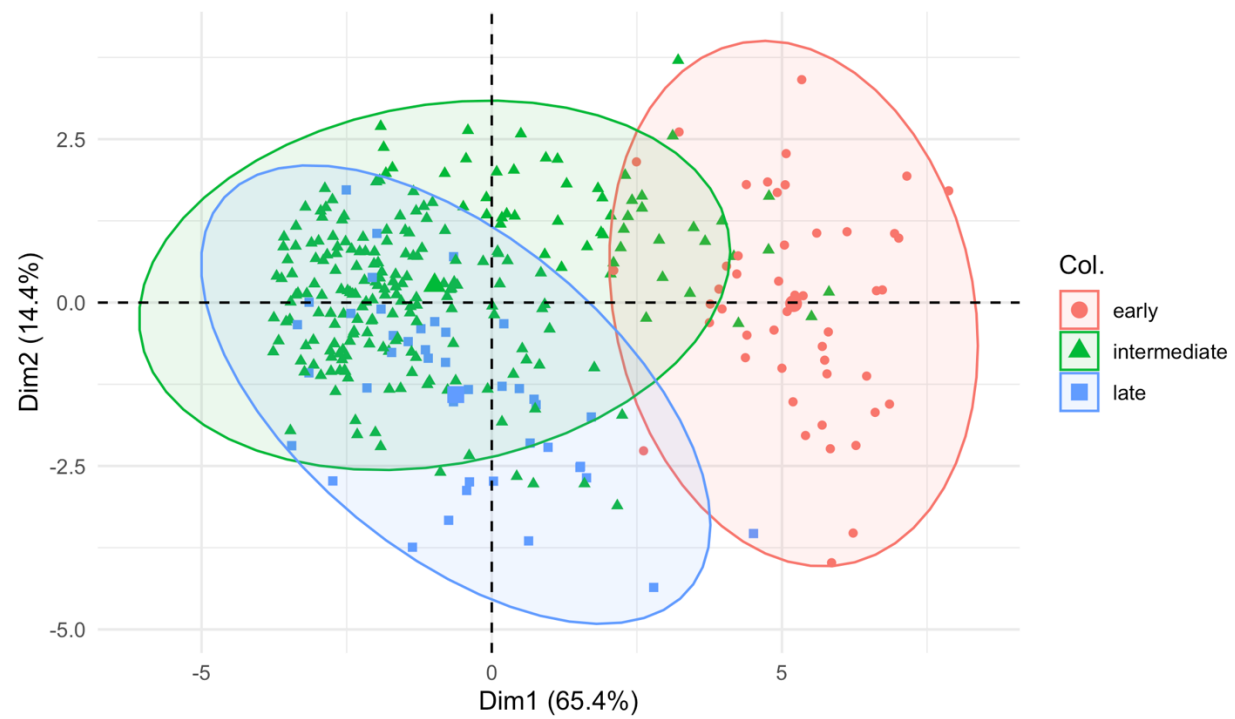

Supplement: Supplementary file 7 — APPENDIX S7. Principal components analysis of V. riparia clustered by developmental stage. [file AJB2-107-676-s007.pdf]

Appendix S9. Principal components analysis of *V. amurensis* clustered by developmental stage.

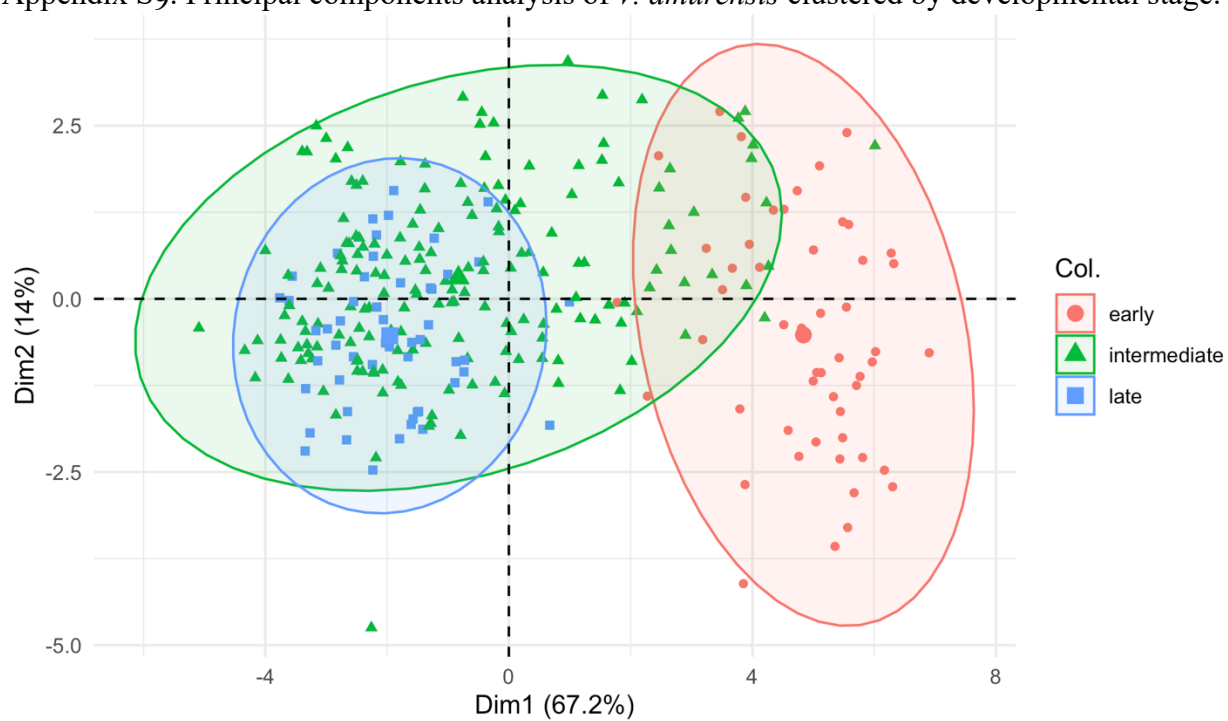

Supplement: Supplementary file 9 — APPENDIX S9. Principal components analysis of V. amurensis clustered by developmental stage. [file AJB2-107-676-s009.pdf]

Appendix S11. Principal components analysis of *Vitis* clustered by species.

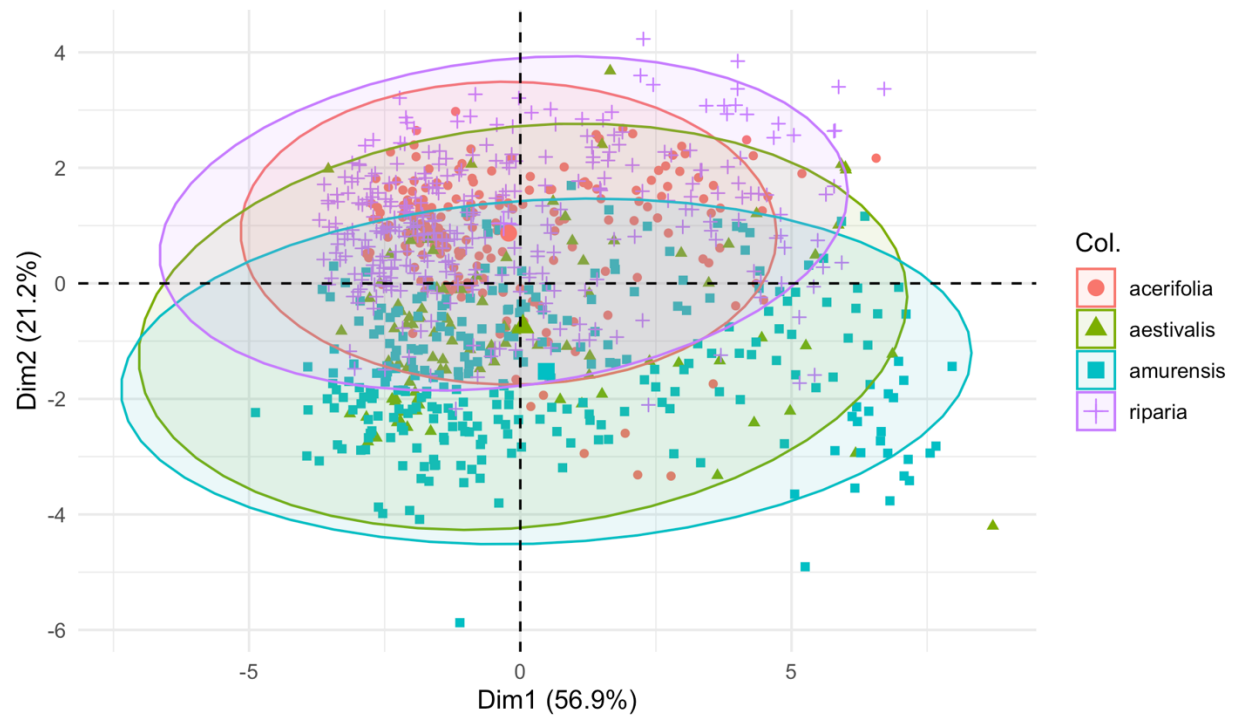

Supplement: Supplementary file 11 — APPENDIX S11. Principal components analysis of Vitis clustered by species. [file AJB2-107-676-s011.pdf]
