# Supplementary material for: The influences of environmental change and development on leaf shape in Vitis
Source: Am J Bot. 2020 Apr 9;107(4):676–88. doi: 10.1002/ajb2.1460 (PMC7217169; doi:10.1002/ajb2.1460)
Supplement: Supplementary file 4 — APPENDIX S4. Loadings of the principal components for the first five dimensions for V. acerifolia. [file AJB2-107-676-s004.pdf]

Appendix S4. Loadings of the principal components for the first five dimensions for *V. acerifolia*.

**Characters**	**PC 1**	**PC 2**	**PC 3**	**PC 4**	**PC 5**
leaf area	-0.321	0.184	0.090	-0.160	0.068
feret diameter ratio	-0.229	-0.276	0.246	0.424	-0.765
tooth area: perimeter	-0.331	0.109	-0.098	0.145	0.182
tooth area: int. perimeter	-0.311	0.219	0.079	0.229	0.213
average tooth area	-0.331	0.154	-0.116	0.043	0.032
tooth area: blade area	0.160	0.268	-0.565	0.683	0.053
teeth: perimeter	0.319	-0.175	0.105	0.066	0.189
teeth: int.perimeter	0.320	-0.038	0.300	0.159	0.214
perimeter: area	0.339	-0.013	-0.085	0.081	-0.141
perimeter ratio	0.071	0.431	0.683	0.330	0.125
compactness	0.172	0.503	-0.083	-0.244	-0.370
shape factor	-0.182	-0.506	0.019	0.205	0.273
teeth: blade area	0.340	-0.083	-0.005	0.077	-0.003
