# Supplementary material for: The influences of environmental change and development on leaf shape in Vitis
Source: Am J Bot. 2020 Apr 9;107(4):676–88. doi: 10.1002/ajb2.1460 (PMC7217169; doi:10.1002/ajb2.1460)
Supplement: Supplementary file 6 — APPENDIX S6. Loadings of the principal components for the first five dimensions for V. aestivalis. [file AJB2-107-676-s006.pdf]

Appendix S6. Loadings of the principal components for the first five dimensions for *V. aestivalis*.

**Characters**	**PC 1**	**PC 2**	**PC 3**	**PC 4**	**PC 5**
leaf area	-0.329	-0.091	-0.154	-0.063	0.356
feret diameter ratio	-0.081	-0.095	0.005	0.989	0.0579
tooth area: perimeter	-0.319	0.279	0.072	-0.022	-0.032
tooth area: int. perimeter	-0.286	0.381	0.176	-0.008	0.121
average tooth area	-0.333	0.242	-0.059	-0.003	-0.058
tooth area: blade area	0.115	0.614	0.100	0.095	-0.467
teeth: perimeter	0.320	-0.151	0.203	-0.022	0.093
teeth: int.perimeter	0.322	-0.032	0.286	-0.010	0.240
perimeter: area	0.340	0.137	0.002	0.061	-0.254
perimeter ratio	0.152	0.400	0.415	0.033	0.620
compactness	0.234	0.247	-0.558	0.027	0.228
shape factor	-0.234	-0.247	0.558	-0.027	-0.228
teeth: blade area	0.352	0.018	0.091	0.028	-0.116
