# Supplementary material for: The influences of environmental change and development on leaf shape in Vitis
Source: Am J Bot. 2020 Apr 9;107(4):676–88. doi: 10.1002/ajb2.1460 (PMC7217169; doi:10.1002/ajb2.1460)
Supplement: Supplementary file 8 — APPENDIX S8. Loadings of the principal components for the first five dimensions for V. riparia. [file AJB2-107-676-s008.pdf]

Appendix S8. Loadings of the principal components for the first five dimensions for *V. riparia*.

**Characters**	**PC 1**	**PC 2**	**PC 3**	**PC 4**	**PC 5**
leaf area	-0.322	0.179	-0.134	-0.058	0.185
feret diameter ratio	-0.130	-0.017	0.620	-0.772	0.035
tooth area: perimeter	-0.325	0.109	0.173	0.182	-0.010
tooth area: int. perimeter	-0.300	0.240	0.234	0.231	0.098
average tooth area	-0.328	0.179	0.029	0.072	-0.104
tooth area: blade area	0.201	0.188	0.528	0.356	-0.583
teeth: perimeter	0.312	-0.182	0.107	0.037	0.288
teeth: int.perimeter	0.315	-0.051	0.164	0.088	0.396
perimeter: area	0.336	-0.018	0.052	-0.018	-0.194
perimeter ratio	0.148	0.509	0.268	0.217	0.542
compactness	0.212	0.513	-0.239	-0.251	-0.130
shape factor	-0.212	-0.513	0.239	0.251	0.130
teeth: blade area	0.338	-0.091	0.078	0.006	-0.003
