# Supplementary material for: The influences of environmental change and development on leaf shape in Vitis
Source: Am J Bot. 2020 Apr 9;107(4):676–88. doi: 10.1002/ajb2.1460 (PMC7217169; doi:10.1002/ajb2.1460)
Supplement: Supplementary file 10 — APPENDIX S10. Loadings of the principal components for the first five dimensions for V. amurensis. [file AJB2-107-676-s010.pdf]

Appendix S10. Loadings of the principal components for the first five dimensions for *V. amurensis*.

Characters	PC 1	PC 2	PC 3	PC 4	PC 5
leaf area	-0.305	0.159	0.073	-0.393	-0.085
feret diameter ratio	-0.056	-0.027	0.967	0.237	-0.065
tooth area: perimeter	-0.316	0.170	-0.072	0.252	0.113
tooth area: int. perimeter	-0.294	0.295	-0.045	0.221	0.273
average tooth area	-0.320	0.176	-0.083	0.184	0.001
tooth area: blade area	0.262	0.127	-0.139	0.663	0.004
teeth: perimeter	0.319	-0.157	0.046	-0.164	0.154
teeth: int.perimeter	0.323	-0.052	0.063	-0.180	0.272
perimeter: area	0.331	-0.013	0.025	-0.019	-0.104
perimeter ratio	0.172	0.503	0.124	-0.179	0.697
compactness	0.228	0.508	0.013	-0.064	-0.382
shape factor	-0.228	-0.508	-0.013	0.064	0.382
teeth: blade area	0.312	-0.115	-0.066	0.323	0.120
