# Supplementary material for: The influences of environmental change and development on leaf shape in Vitis
Source: Am J Bot. 2020 Apr 9;107(4):676–88. doi: 10.1002/ajb2.1460 (PMC7217169; doi:10.1002/ajb2.1460)
Supplement: Supplementary file 12 — APPENDIX S12. Loadings of the principal components for the first five dimensions for Vitis. [file AJB2-107-676-s012.pdf]

Appendix S12. Loadings of the principal components for the first five dimensions for *Vitis*.

**Characters**	**PC 1**	**PC 2**	**PC 3**	**PC 4**	**PC 5**
leaf area	-0.339	-0.002	-0.121	-0.267	0.257
feret diameter ratio	-0.093	-0.107	0.818	-0.503	-0.241
tooth area: perimeter	-0.344	0.137	0.113	0.206	0.033
tooth area: int. perimeter	-0.315	0.235	0.182	0.212	0.190
average tooth area	-0.349	0.158	0.022	0.100	-0.014
tooth area: blade area	0.128	0.409	0.281	0.520	-0.365
teeth: perimeter	0.316	-0.245	0.093	0.042	0.240
teeth: int.perimeter	0.327	-0.149	0.154	0.051	0.374
perimeter: area	0.344	0.142	0.043	0.035	-0.192
perimeter ratio	0.121	0.401	0.300	0.046	0.680
compactness	0.165	0.477	-0.176	-0.377	-0.065
shape factor	-0.166	-0.479	0.172	0.376	0.053
teeth: blade area	0.355	-0.046	0.084	0.129	-0.047
