# Supplementary material for: The influences of environmental change and development on leaf shape in Vitis
Source: Am J Bot. 2020 Apr 9;107(4):676–88. doi: 10.1002/ajb2.1460 (PMC7217169; doi:10.1002/ajb2.1460)
Supplement: Supplementary file 13 — APPENDIX S13. Bootstrap Forest analysis of Vitis species based on leaf shape. [file AJB2-107-676-s013.pdf]

Appendix S13. Bootstrap Forest analysis of *Vitis* species based on leaf shape.

**Species**	**acerifolia**	**aestivalis**	**amurensis**	**riparia**
acerifolia	194	0	0	10
aestivalis	4	73	5	14
amurensis	1	1	281	2
riparia	3	2	5	307

Note: Misclassification rate was 5.21%.
