# Supplementary material for: The influences of environmental change and development on leaf shape in Vitis
Source: Am J Bot. 2020 Apr 9;107(4):676–88. doi: 10.1002/ajb2.1460 (PMC7217169; doi:10.1002/ajb2.1460)
Supplement: Supplementary file 14 — APPENDIX S14. Bootstrap Forest analysis of Vitis species based on all measured leaf shape characters. [file AJB2-107-676-s014.pdf]

Appendix S14. Bootstrap Forest analysis of *Vitis* species based on all measured leaf shape characters.

**Term**	**G^2**	**Portion**
tooth area: blade area	186.431	0.159
total teeth	166.710	0.142
teeth: perimeter	99.979	0.085
average tooth area	80.004	0.068
teeth: internal perimeter°‡	78.876	0.067
feret diameter ratio°	73.748	0.063
compactness	72.248	0.062
shape factor	70.670	0.060
leaf area‡	66.963	0.057
teeth: blade area	65.971	0.056
tooth area: internal perimeter	60.399	0.052

Note: Only characters with > 0.05 portion included. ° in DiLP MAT equation. ‡ in DiLP MAP equation.
