# Supplementary material for: The influences of environmental change and development on leaf shape in Vitis
Source: Am J Bot. 2020 Apr 9;107(4):676–88. doi: 10.1002/ajb2.1460 (PMC7217169; doi:10.1002/ajb2.1460)
Supplement: Supplementary file 15 — APPENDIX S15. Bootstrap Forest analysis of Vitis acerifolia, Vitis aestivalis, Vitis amurensis, and Vitis riparia by year based on leaf shape. [file AJB2-107-676-s015.pdf]

Appendix S15. Bootstrap Forest analysis of *Vitis acerifolia*, *Vitis aestivalis*, *Vitis amurensis,* and *Vitis riparia* by year based on leaf shape.

**Species**	**Year**	**2013**	**2015**	**Misclassification rate**
V. acerifolia	2013	96	5	4.4%
	2015	5	99	
V. aestivalis	2013	48	2	8.3%
	2015	6	40	
V. amurensis	2013	136	8	8.8%
	2015	17	124	
V. riparia	2013	170	4	4.1%
	2015	9	134	
