# Supplementary material for: The influences of environmental change and development on leaf shape in Vitis
Source: Am J Bot. 2020 Apr 9;107(4):676–88. doi: 10.1002/ajb2.1460 (PMC7217169; doi:10.1002/ajb2.1460)
Supplement: Supplementary file 16 — APPENDIX S16. Bootstrap Forest analysis of Vitis acerifolia, Vitis aestivalis, Vitis amurensis, and Vitis riparia by year based on all measured leaf shape characters. [file AJB2-107-676-s016.pdf]

Appendix S16. Bootstrap Forest analysis of *Vitis acerifolia, Vitis aestivalis, Vitis amurensis*, and *Vitis riparia* by year based on all measured leaf shape characters.

Species	Character	G^2	Proportion
V. acerifolia	tooth area: blade area	11.396	0.167
	total teeth	5.921	0.087
	compactness	5.379	0.079
	perimeter ratio‡	5.305	0.078
	tooth area: perimeter	5.228	0.077
	shape factor	5.144	0.075
	feret diameter ratio°	4.922	0.072
	tooth area: internal perimeter	4.847	0.071
	leaf area‡	4.270	0.063
V. aestivalis	compactness	7.428	0.184
	shape factor	7.356	0.183
	tooth area: blade area	3.182	0.079
	teeth: blade area	3.043	0.076
	tooth area: internal perimeter	2.562	0.064
	feret diameter ratio°	2.426	0.060
	tooth area: perimeter	2.416	0.060
	perimeter: area	2.407	0.060
	perimeter ratio‡	2.340	0.058
V. amurensis	perimeter ratio‡	15.823	0.134
	tooth area: internal perimeter	12.557	0.106
	tooth area: perimeter	11.023	0.093
	total teeth	10.925	0.093
	teeth: perimeter	10.680	0.090
	average tooth area	8.472	0.072
	perimeter: area	7.671	0.065
	compactness	7.277	0.062
	shape factor	6.859	0.058
	teeth: internal perimeter°‡	6.363	0.054
V. riparia	compactness	27.521	0.163
	shape factor	24.100	0.143
	teeth: perimeter	13.492	0.080
	perimeter ratio‡	13.109	0.078
	tooth area: perimeter	12.982	0.077
	tooth area: internal perimeter	12.751	0.076
	teeth: internal perimeter°‡	10.608	0.063
	average tooth area	10.450	0.062
	tooth area: blade area	9.908	0.059
	feret diameter ratio°	8.805	0.052

Note: Only includes characters with > 0.05 proportion. ° denotes variable in DiLP MAT equation. ‡ denotes variable in DiLP MAP equation.
