# Supplementary material for: The influences of environmental change and development on leaf shape in Vitis
Source: Am J Bot. 2020 Apr 9;107(4):676–88. doi: 10.1002/ajb2.1460 (PMC7217169; doi:10.1002/ajb2.1460)
Supplement: Supplementary file 17 — APPENDIX S17. Student's t‐tests of Vitis acerifolia of all measured leaf characters. [file AJB2-107-676-s017.pdf]

Appendix S17. Student's t-tests of *Vitis acerifolia* of all measured leaf characters.

V. acerifolia **Character**	**bin**	**df**	**t value**	**p value**
total teeth	mean	202	-0.664	0.508
	1	5	-0.799	0.460
	2	183	-0.634	0.527
	3	10	0.643	0.534
leaf area	mean	202	0.153	0.878
	1	5	-1.090	0.325
	2	183	0.048	0.962
	3	10	-0.350	0.734
feret diameter ratio	mean	202	-0.540	0.590
	1	5	-0.415	0.695
	2	183	-1.214	0.226
	3	10	-0.221	0.829
average tooth area	mean	201	-0.064	0.949
	1	5	-1.411	0.217
	2	182	-0.054	0.957
	3	10	-1.004	0.339
tooth area: perimeter	mean	201	-0.550	0.583
	1	5	-1.316	0.245
	2	182	-0.681	0.497
	3	10	-0.688	0.507
tooth area: internal perimeter	mean	201	-0.704	0.482
	1	5	-1.062	0.337
	2	182	-0.841	0.402
	3	10	-0.423	0.681
tooth area: blade area	mean	201	-2.749	0.007*
	1	5	-4.800	0.005*
	2	182	-2.579	0.011*
	3	10	0.219	0.831
teeth: perimeter	mean	202	-0.621	0.535
	1	5	1.029	0.351
	2	183	-0.675	0.501
	3	10	1.074	0.308
teeth: internal perimeter	mean	202	-0.724	0.47
	1	5	1.395	0.222
	2	183	-0.757	0.450
	3	10	1.362	0.203
teeth: blade area	mean	202	-1.111	0.268
	1	5	0.525	0.622
	2	183	-0.989	0.324
	3	10	1.280	0.230
perimeter: area	mean	202	-1.100	0.273
	1	5	0.219	0.835

	2	183	-0.865	0.388
	3	10	1.312	0.219
perimeter ratio	mean	202	-0.583	0.561
	1	5	-0.249	0.813
	2	183	-0.541	0.589
	3	10	0.678	0.514
compactness	mean	202	-1.233	0.219
	1	5	-1.941	0.11
	2	183	-0.737	0.462
	3	10	0.519	0.615
shape factor	mean	202	0.367	0.714
	1	5	2.106	0.089
	2	183	-0.124	0.902
	3	10	-0.117	0.909

Note: * denotes p value of < 0.05.
