# Supplementary material for: The influences of environmental change and development on leaf shape in Vitis
Source: Am J Bot. 2020 Apr 9;107(4):676–88. doi: 10.1002/ajb2.1460 (PMC7217169; doi:10.1002/ajb2.1460)
Supplement: Supplementary file 18 — APPENDIX S18. Student's t‐tests of Vitis aestivalis of all measured leaf characters. [file AJB2-107-676-s018.pdf]

Appendix S18. Student's t-tests of *Vitis aestivalis* of all measured leaf characters.

V. amurensis **Character**	**bin**	**df**	**t value**	**p value**
total teeth	mean	94	-2.637	0.010*
	1	1	-16.166	0.039*
	2	80	-2.553	0.013*
	3	9	-0.563	0.588
leaf area	mean	94	0.326	0.745
	1	1	-0.550	0.680
	2	80	0.508	0.613
	3	9	-0.765	0.464
feret diameter ratio	mean	93	0.573	0.568
	1	1	-1.476	0.379
	2	79	0.543	0.589
	3	9	-0.219	0.832
average tooth area	mean	93	-0.293	0.770
	1	1	-0.301	0.814
	2	79	-0.047	0.963
	3	9	-1.645	0.134
tooth area: perimeter	mean	93	-0.378	0.706
	1	1	-0.716	0.604
	2	79	-0.053	0.958
	3	9	-1.576	0.150
tooth area: internal perimeter	mean	93	-0.807	0.422
	1	1	-0.964	0.512
	2	79	-0.375	0.709
	3	9	-1.430	0.187
tooth area: blade area	mean	93	-2.586	0.011*
	1	1	-30.217	0.021*
	2	79	-2.379	0.020*
	3	9	-0.812	0.438
teeth: perimeter	mean	94	-0.633	0.528
	1	1	-0.477	0.717
	2	80	-0.449	0.654
	3	9	1.169	0.273
teeth: internal perimeter	mean	94	-0.851	0.397
	1	1	-0.568	0.671
	2	80	-0.541	0.59
	3	9	1.125	0.290
teeth: blade area	mean	94	-1.134	0.260
	1	1	-0.344	0.789
	2	80	-0.899	0.372
	3	9	1.182	0.268
perimeter: area	mean	94	-1.257	0.212
	1	1	0.184	0.884

	2	80	-1.300	0.197
	3	9	0.991	0.348
perimeter ratio	mean	94	-1.494	0.139
	1	1	-1.743	0.332
	2	80	-0.934	0.353
	3	9	-0.283	0.784
compactness	mean	94	-4.333	3.689e-05*
	1	1	-1.996	0.296
	2	80	-4.089	0.0001*
	3	9	-0.185	0.857
shape factor	mean	94	4.670	1.002e-05*
	1	1	2.922	0.210
	2	80	4.628	1.405e-05*
	3	9	0.380	0.713

Note: * denotes p value of < 0.05.
