# Supplementary material for: The influences of environmental change and development on leaf shape in Vitis
Source: Am J Bot. 2020 Apr 9;107(4):676–88. doi: 10.1002/ajb2.1460 (PMC7217169; doi:10.1002/ajb2.1460)
Supplement: Supplementary file 19 — APPENDIX S19. Student's t‐tests of Vitis amurensis of all measured leaf characters. [file AJB2-107-676-s019.pdf]

Appendix S19. Student's t-tests of *Vitis amurensis* of all measured leaf characters.

V. amurensis **Character**	**bin**	**df**	**t value**	**p value**
total teeth	mean	283	-4.005	7.948e-05*
	1	50	-4.982	7.904e-06*
	2	160	-2.677	0.008*
	3	30	-0.916	0.367
leaf area	mean	205	-1.924	0.056
	1	46	-2.795	0.008*
	2	109	-2.320	0.022*
	3	22	-0.740	0.467
feret diameter ratio	mean	204	0.480	0.632
	1	46	-1.008	0.319
	2	109	0.599	0.551
	3	22	0.261	0.796
average tooth area	mean	283	3.775	0.0002*
	1	50	-2.256	0.03*
	2	160	-3.743	0.0003*
	3	30	-1.676	0.104
tooth area: perimeter	mean	281	-3.307	0.001*
	1	50	-1.773	0.082
	2	158	-3.61	0.0004*
	3	30	-1.992	0.056
tooth area: internal perimeter	mean	282	-4.400	1.54e-05*
	1	50	-2.604	0.012*
	2	159	-4.505	1.279e-05*
	3	30	-3.042	0.005*
tooth area: blade area	mean	205	0.811	0.418
	1	46	2.395	0.021*
	2	109	0.508	0.613
	3	22	-0.591	0.561
teeth: perimeter	mean	281	1.902	0.058
	1	50	1.890	0.065
	2	158	3.236	0.001*
	3	30	1.146	0.261
teeth: internal perimeter	mean	282	1.018	0.310
	1	50	0.336	0.738
	2	159	2.362	0.019*
	3	30	-0.079	0.938
teeth: blade area	mean	205	1.285	0.200
	1	46	2.624	0.012*
	2	109	2.335	0.021*
	3	22	0.028	0.978
perimeter: area	mean	280	1.892	0.059
	1	50	2.751	0.008*

	2	157	2.574	0.011*
	3	30	0.737	0.467
perimeter ratio	mean	280	-3.979	8.838e-05*
	1	50	-4.286	8.276e-05*
	2	157	-1.754	0.081
	3	30	-3.153	0.004*
compactness	mean	280	-1.932	0.054
	1	50	-2.219	0.031*
	2	157	-0.409	0.683
	3	30	-1.210	0.236
shape factor	mean	280	2.446	0.015*
	1	50	2.568	0.013*
	2	157	0.989	0.324
	3	30	1.429	0.163

Note: * denotes p value of < 0.05.
