# Supplementary material for: The influences of environmental change and development on leaf shape in Vitis
Source: Am J Bot. 2020 Apr 9;107(4):676–88. doi: 10.1002/ajb2.1460 (PMC7217169; doi:10.1002/ajb2.1460)
Supplement: Supplementary file 20 — APPENDIX S20. Student's t‐tests of Vitis riparia of all measured leaf characters. [file AJB2-107-676-s020.pdf]

Appendix S20. Student's t-tests of *Vitis riparia* of all measured leaf characters.

V. riparia **Character**	**bin**	**df**	**t value**	**p value**
total teeth	mean	315	-0.845	0.399
	1	45	-0.741	0.463
	2	227	1.070	0.286
	3	39	0.178	0.864
leaf area	mean	315	-1.383	0.168
	1	45	0.298	0.767
	2	227	-0.009	0.993
	3	39	-1.108	0.275
feret diameter ratio	mean	315	0.209	0.835
	1	45	0.836	0.407
	2	227	0.025	0.980
	3	39	-1.610	0.115
average tooth area	mean	313	-0.093	0.926
	1	44	-0.123	0.903
	2	226	1.349	0.179
	3	39	-0.860	0.395
tooth area: perimeter	mean	313	1.631	0.104
	1	44	2.316	0.025*
	2	226	2.681	0.008*
	3	39	0.718	0.477
tooth area: internal perimeter	mean	311	2.328	0.021*
	1	43	1.506	0.139
	2	225	3.795	0.0002*
	3	39	0.970	0.338
tooth area: blade area	mean	313	2.556	0.011*
	1	44	-0.272	0.787
	2	226	1.35	0.178
	3	39	1.734	0.091
teeth: perimeter	mean	315	2.971	0.003*
	1	45	2.519	0.015*
	2	227	2.210	0.028*
	3	39	1.210	0.234
teeth: internal perimeter	mean	313	3.213	0.001*
	1	44	2.318	0.025*
	2	226	3.077	0.002*
	3	39	1.424	0.162
teeth: blade area	mean	314	1.237	0.217
	1	44	0.241	0.811
	2	227	0.504	0.615
	3	39	0.605	0.549
perimeter: area	mean	315	-0.752	0.453
	1	45	-3.392	0.001*

	2	227	-0.888	0.376
	3	39	0.071	0.944
perimeter ratio	mean	313	2.239	0.026*
	1	44	0.046	0.964
	2	226	3.587	0.0004*
	3	39	1.687	0.100
compactness	mean	315	-5.818	1.464e-08*
	1	45	-7.651	1.117e-09*
	2	227	-3.786	0.0002*
	3	39	-1.357	0.183
shape factor	mean	315	5.581	5.171e-08*
	1	45	7.566	1.488e-09*
	2	227	3.869	0.0001*
	3	39	1.623	0.113

Note: * denotes p value of < 0.05.
