# Supplementary material for: The influences of environmental change and development on leaf shape in Vitis
Source: Am J Bot. 2020 Apr 9;107(4):676–88. doi: 10.1002/ajb2.1460 (PMC7217169; doi:10.1002/ajb2.1460)
Supplement: Supplementary file 21 — APPENDIX S21. Linear model of Vitis acerifolia based on all measured leaf shape characters. [file AJB2-107-676-s021.pdf]

Appendix S21. Linear model of *Vitis acerifolia* based on all measured leaf shape characters.

V. acerifolia							
**Year**	**Character**	**Coefficient**	**Estimate**	**Std Error**	**t value**	**p value**	**Adjusted R^2**
combined	total teeth	Intercept	48.758	1.173	41.584	< 2e-16	0.201
		leaf	-1.191	0.165	-7.205	1.13e-11	
combined	feret diameter ratio	Intercept	0.730	0.005	164.09	< 2e-16	**0.493**
		leaf	0.009	0.001	14.08	< 2e-16	
combined	average tooth area	Intercept	0.053	0.008	6.305	1.80e-09	0.183
		leaf	0.008	0.001	6.801	1.16e-10	
combined	tooth area: perimeter	Intercept	0.042	0.003	13.009	< 2e-16	0.207
		leaf	0.003	0.0004	7.333	5.42e-12	
combined	tooth area: internal perimeter	Intercept	0.065	0.005	13.455	< 2e-16	0.122
		leaf	0.004	0.001	5.401	1.86e-07	
2012-2013	tooth area: blade area	Intercept	0.070	0.002	32.325	< 2e-16	0.013
		leaf	-0.0004	0.0003	-1.509	0.135	
2014-2015		Intercept	0.080	0.002	35.601	< 2e-16	0.168
		leaf	-0.002	0.0003	-4.653	9.95e-06	
combined	teeth: perimeter	Intercept	1.083	0.050	21.683	< 2e-16	0.141
		leaf	-0.041	0.007	-5.857	1.88e-08	
combined	teeth: internal perimeter	Intercept	1.658	0.073	22.784	< 2e-16	0.224
		leaf	-0.079	0.010	-7.725	5.11e-13	
combined	teeth: blade area	Intercept	2.691	0.216	12.447	< 2e-16	0.193
		leaf	-0.214	0.031	-7.037	2.99e-11	
combined	perimeter: area	Intercept	2.167	0.110	19.723	< 2e-16	0.260
		leaf	-0.132	0.016	-8.508	3.96e-15	
combined	perimeter ratio	Intercept	1.533	0.022	70.576	< 2e-16	0.183
		leaf	-0.021	0.003	-6.817	1.05e-10	
combined	compactness	Intercept	91.337	2.252	40.57	< 2e-16	**0.479**
		leaf	-4.349	0.317	-13.70	< 2e-16	
combined	shape factor	Intercept	0.125	0.007	17.39	< 2e-16	**0.486**
		leaf	0.014	0.001	13.89	< 2e-16	

Note: Bold text denotes $R^2 \geqslant 0.3$.
