# Supplementary material for: The influences of environmental change and development on leaf shape in Vitis
Source: Am J Bot. 2020 Apr 9;107(4):676–88. doi: 10.1002/ajb2.1460 (PMC7217169; doi:10.1002/ajb2.1460)
Supplement: Supplementary file 22 — APPENDIX S22. Breakpoint analysis of Vitis acerifolia based on all measured leaf shape characters. [file AJB2-107-676-s022.pdf]

Appendix S22. Breakpoint analysis of *Vitis acerifolia* based on all measured leaf shape characters.

Year	Character	BP 1	Std Err	BP 2	Std Err
combined	total teeth	2.087	0.482	9.409	1.441
combined	feret diameter ratio	4.207	0.369	9.644	0.660
combined	average tooth area	3.143	1.056	10.631	1.494
combined	tooth area: perimeter	6.215	0.562	10.660	0.638
combined	tooth area: internal perimeter	4.657	3.754	10.771	1.104
2012-2013	tooth area: blade area	2.838	1.032	10.931	0.449
2014-2015		4.705	0.824	10.666	0.840
combined	teeth: perimeter	4.470	0.589	10.971	0.560
combined	teeth: internal perimeter	2.237	0.340	6.583	0.777
combined	teeth: blade area	4.321	0.423	10.939	0.857
combined	perimeter: area	2.540	0.916	6.566	0.677
combined	perimeter ratio	2.347	0.383	10.212	9.734
combined	compactness	2.835	0.663	10.758	7.775
combined	shape factor	2.856	0.896	9.729	1.593

Note: Separate breakpoint analyses were performed for characters with statistical differences between leaf-growing seasons.
