# Supplementary material for: The influences of environmental change and development on leaf shape in Vitis
Source: Am J Bot. 2020 Apr 9;107(4):676–88. doi: 10.1002/ajb2.1460 (PMC7217169; doi:10.1002/ajb2.1460)
Supplement: Supplementary file 23 — APPENDIX S23. Linear model of Vitis aestivalis based on all measured leaf shape characters. [file AJB2-107-676-s023.pdf]

Appendix S23. Linear model of *Vitis aestivalis* based on all measured leaf shape characters.

V. aestivalis							
**Year**	**Character**	**Coefficients**	**Estimate**	**Std Error**	**t value**	**p value**	**Adjusted R^2**
2012-2013	total teeth	Intercept	66.974	6.481	10.335	8.53e-14	0.033
		leaf	-1.400	0.857	-1.633	0.109	
2014-2015		Intercept	74.833	7.239	10.338	2.37e-13	0.002
		leaf	-1.117	1.080	-1.034	0.307	
combined	feret diameter ratio	Intercept	0.686	0.024	28.928	< 2e-16	0.108
		leaf	0.012	0.003	3.512	0.0007	
combined	average tooth area	Intercept	0.032	0.014	2.393	0.019	0.103
		leaf	0.006	0.002	3.434	0.0009	
combined	tooth area: perimeter	Intercept	0.030	0.005	6.063	2.84e-08	0.182
		leaf	0.003	0.0007	4.677	9.85e-06	
combined	tooth area: internal perimeter	Intercept	0.044	0.007	6.566	2.92e-09	0.077
		leaf	0.003	0.001	2.974	0.004	
2012-2013	tooth area: blade area	Intercept	0.047	0.005	9.151	5.17e-12	0.025
		leaf	-0.001	0.0007	-1.501	0.14	
2014-2015		Intercept	0.070	0.007	9.539	2.81e-12	0.152
		leaf	-0.003	0.001	-3.015	0.004	
combined	teeth: perimeter	Intercept	2.179	0.145	15.035	< 2e-16	**0.388**
		leaf	-0.158	0.020	-7.825	7.41e-12	
combined	teeth: internal perimeter	Intercept	3.019	0.192	15.742	< 2e-16	**0.445**
		leaf	-0.238	0.027	-8.872	4.6e-14	
combined	teeth: blade area	Intercept	6.556	0.672	9.764	5.84e-16	**0.354**
		leaf	-0.683	0.094	-7.284	9.85e-11	
combined	perimeter: area	Intercept	2.779	0.220	12.614	< 2e-16	**0.374**
		leaf	-0.234	0.031	-7.602	2.17e-11	

combined	perimeter ratio	Intercept	1.450	0.032	44.707	< 2e-16	**0.323**
		leaf	-0.031	0.005	-6.802	9.47e-10	
2012-2013	compactness	Intercept	56.834	4.223	13.46	< 2e-16	0.048
		leaf	-1.039	0.559	-1.86	0.069	
2014-2015		Intercept	107.088	7.843	13.655	< 2e-16	**0.379**
		leaf	-6.247	1.170	-5.338	3.13e-06	
2012-2013	shape factor	Intercept	0.230	0.022	10.589	3.76e-14	0.049
		leaf	0.005	0.003	1.878	0.067	
2014-2015		Intercept	0.119	0.017	6.999	1.15e-08	**0.415**
		leaf	0.015	0.003	5.734	8.30e-07	

Note: Bold text denotes $R^2 \geqslant 0.3$.
