# Supplementary material for: The influences of environmental change and development on leaf shape in Vitis
Source: Am J Bot. 2020 Apr 9;107(4):676–88. doi: 10.1002/ajb2.1460 (PMC7217169; doi:10.1002/ajb2.1460)
Supplement: Supplementary file 24 — APPENDIX S24. Breakpoint analysis of Vitis aestivalis based on all measured leaf shape characters. [file AJB2-107-676-s024.pdf]

Appendix S24. Breakpoint analysis of *Vitis aestivalis* based on all measured leaf shape characters.

Year	Character	BP 1	Std Err	BP 2	Std Err
2012-2013	total teeth	3.368	0.693	8.740	1.382
2014-2015		2.345	0.587	8.160	1.099
combined	feret diameter ratio	3.518	0.981	10.181	2.338
combined	average tooth area	4.682	2.400	9.021	1.613
combined	tooth area: perimeter	2.149	0.591	10.612	9.157
combined	tooth area: internal perimeter	2.040	0.515	9.062	0.846
2012-2013	tooth area: blade area	3.639	0.805	9.413	0.894
2013-2014		2.006	0.430	8.662	1.919
combined	teeth: perimeter	2.025	0.144	9.801	1.535
combined	teeth: internal perimeter	2.008	0.173	9.691	1.474
combined	teeth: blade area	2	0.125	6	1.989
combined	perimeter: area	2.120	0.300	9.991	0.670
combined	perimeter ratio	2.271	0.744	10.033	1.335
2012-2013	compactness	5.783	0.706	10.479	0.988
2014-2015		2.524	0.795	9.153	3.350
2012-2013	shape factor	3.670	2.563	10.239	1.830
2014-2015		2.302	2.353	10.689	2.776

Note: Separate breakpoint analyses were performed for characters with statistical differences between leaf-growing seasons.
