# Supplementary material for: The influences of environmental change and development on leaf shape in Vitis
Source: Am J Bot. 2020 Apr 9;107(4):676–88. doi: 10.1002/ajb2.1460 (PMC7217169; doi:10.1002/ajb2.1460)
Supplement: Supplementary file 25 — APPENDIX S25. Linear model of Vitis amurensis based on all measured leaf shape characters. Bold text denotes R2 ≥ 0.3. [file AJB2-107-676-s025.pdf]

Appendix S25. Linear model of *Vitis amurensis* based on all measured leaf shape characters. Bold text denotes $R^2 \geq 0.3$.

V. amurensis Year	Character	Coefficients	Estimate	Std Error	t value	p value	Adjusted R^2
2012-2013	total teeth	Intercept	66.916	2.880	23.231	< 2e-16	-0.007
		leaf	0.019	0.382	0.051	0.96	
2014-2015		Intercept	80.404	2.913	27.607	< 2e-16	0.022
		leaf	-0.828	0.405	-2.041	0.043	
combined	feret diameter ratio	Intercept	0.761	0.009	85.341	< 2e-16	0.163
		leaf	0.008	0.001	6.406	1.01e-09	
2012-2013	average tooth area	Intercept	0.005	0.008	0.656	0.513	**0.382**
		leaf	0.010	0.001	9.455	< 2e-16	
2014-2015		Intercept	0.012	0.013	0.911	0.364	**0.3450**
		leaf	0.016	0.002	8.730	7.15e-15	
2012-2013	tooth area: perimeter	Intercept	0.020	0.003	7.513	5.91e-12	**0.543**
		leaf	0.005	0.0003	13.073	< 2e-16	
2014-2015		Intercept	0.023	0.004	6.203	6.12e-09	**0.489**
		leaf	0.006	0.0005	11.543	< 2e-16	
2012-2013	tooth area: internal perimeter	Intercept	0.030	0.003	8.594	1.38e-14	**0.425**
		leaf	0.005	0.0005	10.319	< 2e-16	
2014-2015		Intercept	0.039	0.005	8.131	2.19e-13	**0.394**
		leaf	0.006	0.0007	9.561	< 2e-16	
combined	tooth area: blade area	Intercept	0.066	0.002	31.31	< 2e-16	**0.330**
		leaf	-0.003	0.0003	-10.11	< 2e-16	
combined	teeth: perimeter	Intercept	3.726	0.114	32.70	< 2e-16	**0.547**
		leaf	-0.286	0.015	-18.48	< 2e-16	

combined	teeth: internal perimeter	Intercept	5.226	0.149	35.15	< 2e-16	**0.601**
		leaf	-0.417	0.020	-20.67	< 2e-16	
combined	teeth: blade area	Intercept	14.442	0.850	17.00	< 2e-16	**0.432**
		leaf	-1.492	0.119	-12.56	< 2e-16	
combined	perimeter: area	Intercept	3.185	0.126	25.38	< 2e-16	**0.489**
		leaf	-0.280	0.017	-16.44	< 2e-16	
2012-2013	perimeter ratio	Intercept	1.403	0.023	61.621	< 2e-16	0.220
		leaf	-0.019	0.003	-6.421	1.9e-09	
2014-2015		Intercept	1.539	0.021	72.83	< 2e-16	**0.429**
		leaf	-0.030	0.003	-10.19	< 2e-16	
combined	compactness	Intercept	57.660	1.534	37.59	< 2e-16	**0.301**
		leaf	-2.299	0.208	-11.04	< 2e-16	
2012-2013	shape factor	Intercept	0.240	0.013	18.320	< 2e-16	**0.337**
		leaf	0.015	0.002	8.589	1.42e-14	
2014-2015		Intercept	0.212	0.013	16.103	< 2e-16	**0.348**
		leaf	0.016	0.002	8.609	1.61e-14	

Note: Bold text denotes $R^2 \geqslant 0.3$.
