# Supplementary material for: The influences of environmental change and development on leaf shape in Vitis
Source: Am J Bot. 2020 Apr 9;107(4):676–88. doi: 10.1002/ajb2.1460 (PMC7217169; doi:10.1002/ajb2.1460)
Supplement: Supplementary file 26 — APPENDIX S26. Breakpoint analysis of Vitis amurensis based on all measured leaf shape characters. [file AJB2-107-676-s026.pdf]

Appendix S26. Breakpoint analysis of *Vitis amurensis* based on all measured leaf shape characters.

Year	Character	BP 1	Std Err	BP 2	Std Err
2012-2013	total teeth	3.452	0.479	11.542	2.206
2014-2015		3.271	1.045	10.741	2.278
combined	feret diameter ratio	2.192	0.608	7.713	2.891
2012-2013	average tooth area	3.695	0.832	11.318	0.886
2014-2015		2.163	0.622	11.163	2.476
2012-2013	tooth area: perimeter	2.919	1.552	11.172	1.129
2014-2015		2.548	1.406	8.482	0.435
2012-2013	tooth area: internal perimeter	3.042	6.128	10.991	1.010
2014-2015		6.519	1.909	11.299	2.547
combined	tooth area: blade area	2.608	2.248	8.097	1.429
combined	teeth: perimeter	3.256	0.332	11.409	1.066
combined	teeth: internal perimeter	3.560	0.461	9.667	1.195
combined	teeth: blade area	2.521	0.282	6.393	2.474
combined	perimeter: area	3.667	0.189	9.905	3.937
2012-2013	perimeter ratio	2.575	0.289	9.899	1.569
2014-2015		2.315	0.289	9.376	1.039
combined	compactness	2.680	0.337	9.391	80.437
2012-2013	shape factor	2.434	0.355	9.037	91.685
2014-2015		2.510	0.881	12.000	1.518

Note: Separate breakpoint analyses were performed for characters with statistical differences between leaf-growing seasons.
