# Supplementary material for: The influences of environmental change and development on leaf shape in Vitis
Source: Am J Bot. 2020 Apr 9;107(4):676–88. doi: 10.1002/ajb2.1460 (PMC7217169; doi:10.1002/ajb2.1460)
Supplement: Supplementary file 27 — APPENDIX S27. Linear model of Vitis riparia based on all measured leaf shape characters. [file AJB2-107-676-s027.pdf]

Appendix S27. Linear model of *Vitis riparia* based on all measured leaf shape characters.

V. riparia **Year**	**Character**	**Coefficients**	**Estimate**	**Std Error**	**t value**	**p value**	**Adjusted R^2**
combined	total teeth	Intercept	53.920	1.147	47.02	< 2e-16	0.239
		leaf	-1.513	0.151	-10.01	< 2e-16	
combined	feret diameter ratio	Intercept	0.712	0.008	83.895	< 2e-16	0.151
		leaf	0.008	0.001	7.561	4.42e-13	
combined	average tooth area	Intercept	0.073	0.010	7.118	7.51e-12	0.157
		leaf	0.010	0.001	7.702	1.77e-13	
combined	tooth area: perimeter	Intercept	0.046	0.003	14.606	< 2e-16	0.236
		leaf	0.004	0.0004	9.899	< 2e-16	
2012-2013	tooth area: internal perimeter	Intercept	0.080	0.006	12.72	< 2e-16	0.12
		leaf	0.004	0.0008	4.96	1.68e-06	
2014-2015		Intercept	0.070	0.007	10.064	< 2e-16	0.119
		leaf	0.004	0.001	4.437	1.86e-05	
2012-2013	tooth area: blade area	Intercept	0.082	0.003	29.626	< 2e-16	0.014
		leaf	-0.0006	0.0003	-1.859	0.0647	
2014-2015		Intercept	0.084	0.003	31.113	< 2e-16	0.136
		leaf	-0.002	0.0004	-4.802	4.02e-06	
2012-2013	teeth: perimeter	Intercept	1.530	0.072	21.129	< 2e-16	**0.301**
		leaf	-0.078	0.009	-8.684	2.86e-15	
2014-2015		Intercept	1.293	0.063	20.394	< 2e-16	**0.336**
		leaf	-0.077	0.009	-8.536	2e-14	
2012-2013		Intercept	2.430	0.114	21.280	< 2e-16	**0.360**

	teeth: internal	leaf	-0.141	0.014	-9.908	< 2e-16	
2014-2015	perimeter	Intercept	1.991	0.091	21.94	< 2e-16	**0.437**
		leaf	-0.135	0.013	-10.46	< 2e-16	
combined	teeth: blade area	Intercept	4.133	0.240	17.23	< 2e-16	0.290
		leaf	-0.359	0.032	-11.39	< 2e-16	
combined	perimeter: area	Intercept	2.590	0.114	22.78	< 2e-16	0.295
		leaf	-0.173	0.015	-11.54	< 2e-16	
2012-2013	perimeter ratio	Intercept	1.622	0.025	65.355	< 2e-16	0.283
		leaf	-0.026	0.003	-8.332	2.42e-14	
2014-2015		Intercept	1.588	0.023	70.162	< 2e-16	**0.389**
		leaf	-0.030	0.003	-9.498	< 2e-16	
2012-2013	compactness	Intercept	76.568	1.738	44.07	< 2e-16	**0.509**
		leaf	-2.907	0.217	-13.43	< 2e-16	
2014-2015		Intercept	114.733	4.175	27.48	< 2e-16	**0.477**
		leaf	-6.788	0.594	-11.42	< 2e-16	
2012-2013	shape factor	Intercept	0.152	0.008	19.48	< 2e-16	**0.507**
		leaf	0.013	0.001	13.37	< 2e-16	
2014-2015		Intercept	0.110	0.008	13.28	< 2e-16	**0.545**
		leaf	0.015	0.001	13.08	< 2e-16	

Note: Bold text denotes $R^2 \geqslant 0.3$.
