# Supplementary material for: The influences of environmental change and development on leaf shape in Vitis
Source: Am J Bot. 2020 Apr 9;107(4):676–88. doi: 10.1002/ajb2.1460 (PMC7217169; doi:10.1002/ajb2.1460)
Supplement: Supplementary file 28 — APPENDIX S28. Breakpoint analysis of Vitis riparia based on all measured leaf shape characters. [file AJB2-107-676-s028.pdf]

Appendix S28. Breakpoint analysis of *Vitis riparia* based on all measured leaf shape characters.

Year	Character	BP 1	Std Err	BP 2	Std Err
combined	total teeth	4.693	0.931	10.883	1.658
combined	feret diameter ratio	5.659	0.839	10.986	1.667
combined	average tooth area	2.579	0.654	9.422	2.960
combined	tooth area: perimeter	3.158	1.203	12.707	1.463
2012-2013	tooth area: internal perimeter	6.378	0.668	12.554	1.315
2014-2015		6.732	0.329	10.797	1.754
2012-2013	tooth area: blade area	6.135	2.272	10.992	2.178
2014-2015		5.793	0.613	11.763	1.295
2012-2013	teeth: perimeter	3.223	0.318	10.683	0.443
2014-2015		4.367	0.339	12.225	1.868
2012-2013	teeth: internal perimeter	3.460	0.508	10.656	1.809
2014-2015		4.391	0.204	12.152	2.371
combined	teeth: blade area	3.302	0.137	11.167	2.240
combined	perimeter: area	2.904	0.613	7.226	0.577
2012-2013	perimeter ratio	2.010	0.243	10.650	4.386
2014-2015		2.490	0.664	9.696	2.423
2012-2013	compactness	2.465	0.387	12.822	0.897
2014-2015		3.937	0.916	9.968	2.518
2012-2013	shape factor	2.448	0.494	12.899	0.388
2014-2015		6.190	0.703	12.980	0.560

Note: Separate breakpoint analyses were performed for characters with statistical differences between leaf-growing seasons.
